# Analysis of erythrocyte dynamics in Rhesus macaque monkeys during infection with *Plasmodium cynomolgi*

**DOI:** 10.1186/s12936-018-2560-6

**Published:** 2018-11-06

**Authors:** Luis L. Fonseca, Chester J. Joyner, Celia L. Saney, The MaHPIC Consortium, Alberto Moreno, John W. Barnwell, Mary R. Galinski, Eberhard O. Voit

**Affiliations:** 1 The Wallace H. Coulter Department of Biomedical Engineering, Georgia Institute of Technology and Emory University, Atlanta, GA 30332-2000 USA; 2 Malaria Host–Pathogen Interaction Center, Emory Vaccine Center, Yerkes National Primate Research Center, Emory University, Atlanta, GA 30322 USA; 3 Division of Infectious Diseases, Department of Medicine, Emory University, Atlanta, GA 30322 USA; 4 Malaria Branch, Division of Parasitic Diseases and Malaria, Centers for Disease Control and Prevention, Atlanta, GA 30322 USA

**Keywords:** Mathematical model, Host–pathogen interactions, *Macaca mulatta*, *Plasmodium cynomolgi*, *Plasmodium vivax*, Reticulocytes, Anaemia, Zoonosis

## Abstract

**Background:**

Malaria is a major mosquito transmitted, blood-borne parasitic disease that afflicts humans. The disease causes anaemia and other clinical complications, which can lead to death. *Plasmodium vivax* is known for its reticulocyte host cell specificity, but many gaps in disease details remain. Much less is known about the closely related species, *Plasmodium cynomolgi*, although it is naturally acquired and causes zoonotic malaria. Here, a computational model is developed based on longitudinal analyses of *P. cynomolgi* infections in nonhuman primates to investigate the erythrocyte dynamics that is pertinent to understanding both *P. cynomolgi* and *P. vivax* malaria in humans.

**Methods:**

A cohort of five *P. cynomolgi* infected Rhesus macaques (*Macaca mulatta*) is studied, with individuals exhibiting a plethora of clinical outcomes, including varying levels of anaemia. A discrete recursive model with age structure is developed to replicate the dynamics of *P. cynomolgi* blood-stage infections. The model allows for parasitic reticulocyte preference and assumes an age preference among the mature RBCs. RBC senescence is modelled using a hazard function, according to which RBCs have a mean lifespan of 98 ± 21 days.

**Results:**

Based on in vivo data from three cohorts of macaques, the computational model is used to characterize the reticulocyte lifespan in circulation as 24 ± 5 h (n = 15) and the rate of RBC production as 2727 ± 209 cells/h/µL (n = 15). Analysis of the host responses reveals a pre-patency increase in the number of reticulocytes. It also allows the quantification of RBC removal through the bystander effect.

**Conclusions:**

The evident pre-patency increase in reticulocytes is due to a shift towards the release of younger reticulocytes, which could result from a parasite-induced factor meant to increase reticulocyte availability and satisfy the parasite’s tropism, which has an average value of 32:1 in this cohort. The number of RBCs lost due to the bystander effect relative to infection-induced RBC losses is 62% for *P. cynomolgi* infections, which is substantially lower than the value of 95% previously determined for another simian species, *Plasmodium coatneyi*.

**Electronic supplementary material:**

The online version of this article (10.1186/s12936-018-2560-6) contains supplementary material, which is available to authorized users.

## Background

Malaria is a major life-threatening disease caused by parasites of the genus *Plasmodium* [1]. The genus *Plasmodium* includes parasites of different species that can infect humans as well as nonhuman primates (NHPs), rodents, bats, reptiles and birds [2]. *Plasmodium vivax* has a wide geographical distribution and is responsible for almost half of the malarial cases outside of Africa, where *Plasmodium falciparum* predominates [1, 3, 4]. Having been responsible for 8.5 million cases globally in 2016, *P. vivax* constitutes a major challenge towards the goals of the World Health Organization and its partners of eliminating malaria from 35 countries and reducing incidence and mortality rates by 90% by 2030 [1]. Its closely related sister species, *Plasmodium cynomolgi,* is a simian malaria parasite that has been an important model for research [5–8] and is now also recognized as a zoonosis [9].

In the vertebrate host, the infection process begins with a blood-meal by a female *Anopheles* mosquito, which typically results in the inoculation of the host with fewer than 100 sporozoites [10, 11]. Successful sporozoites travel from the skin to the liver, where they infect hepatocytes. From each infected hepatocyte, tens of thousands of merozoites may develop and be released into the bloodstream [12, 13]. Several species including *P. vivax* and *P. cynomolgi* have the additional ability to produce hypnozoites during the liver stage, which are dormant forms of the parasite that may be activated and thus able to cause relapse infections weeks to months after the primary infection [14–17].

During the blood stage of the parasite’s life cycle, merozoites exclusively infect red blood cells (RBCs). The productivity of an infected RBC is much lower than that of an infected hepatocyte, with an infected RBC only producing up to 30 new merozoites, depending on the *Plasmodium* species. In the case of *P. cynomolgi*, the number of released merozoites per infected RBC is on average 16, with a range of 14–20 [18]. At some point during the blood stage cycle, some merozoites become committed to the production of gametocytes, which, if taken up by a mosquito, begin the arthropod stage of the infection.

Whereas the liver stage of the infection proceeds asymptomatically, the blood stage often presents symptoms that are shared by common viral infections, i.e., headaches, fever, chills, dizziness, myalgia, nausea and vomiting [19, 20]. On the other end of the clinical spectrum is severe malaria, which is most often caused by *P. falciparum*, although *P. vivax* may also cause severe disease [21]. Severe malaria complications can develop very rapidly and progress to death within hours or days [22]. Disease manifestations can include, among others, respiratory distress, pulmonary oedema, acute renal failure, thrombocytopaenia, and severe anaemia [23]. With that said, many infections can be asymptomatic, as also shown recently for relapsing [17] and zoonotic cases [9] of *P. cynomolgi*. Both species exhibit tropism toward reticulocytes, which is nearly strict in the case of *P. vivax* and conditional for *P. cynomolgi* [8, 24, 25]. Also, both species produce caveola vesicle complexes in the infected RBCs, which involves remodelling of the host RBC cytoskeleton, and results in increased membrane deformability [24, 26, 27]. And, as mentioned above, both species produce hypnozoites capable of causing relapses.

To characterize and quantify the RBC dynamics during malaria, various mathematical models have been developed with the particular goal of deconvolving and quantifying the different processes of RBC removal. Models of the malarial host–pathogen interactions have been proposed since the late 1980s [28] (reviewed in [29]). Dynamic models for such a purpose are often formulated as sets of ordinary differential equations (ODEs), and in their simplest form are commonly represented with three compartments, namely, RBCs, infected RBCs, and either merozoites or some marker of the immune response [30–33]. More complex models may contain more than three compartments, especially when they focus on antigenic variation, where many parasite variants are considered, and specific and cross-reactive immune responses are included [29]. Attempts have also been made to model the delays inherent to this system, in which case it was necessary to use delayed differential equations, age-structured ODEs, partial differential equations, or discrete implementations of their continuous analogs. All these approaches have advantages and drawbacks [34] that should be taken into consideration, depending on the ultimate goals of the model, i.e., whether the model was developed as a tool for further analytical investigations, data fitting, hypothesis generation, or other purposes.

Here, a time-dependent discrete recursive equation (DRE) model with age structure is used. The model has four compartments: reticulocytes, mature RBCs, infected RBCs and merozoites, all of which have an age structure, except for the merozoites. Unlike most other models [29], the processes here are quantified time-dependently rather than imposing certain kinetic formulations, such as a mass-action representation. This strategy was used to assess the reticulocyte maturation time in circulation, loss of RBCs, and the impact of the immune response. It also permits the time-dependent quantification of the extent of RBC production and of RBC loss through different processes (random death, senescence, parasitization, or bystander effect), as well as an assessment of the reticulocyte timespan in circulation and of the immune response. The result is in each case a personalized model of each macaque’s response to the infection, which is then analysed a posteriori.

The experimental data used in this work were generated from a longitudinal study involving a cohort of Rhesus macaques that were infected with *P. cynomolgi* (B/M strain) sporozoites and followed for 100 days, with sampling of blood and bone marrow at different points in time. Of particular importance for the model, complete blood counts (CBCs) and parasitaemia counts were performed. The data are publicly available [35], and a comprehensive clinical analysis of these infections [17], as well as multi-omic integrated analyses have been reported [36].

## Methods

### Model formulation

A dynamic mathematical model was developed to characterize the within-host host–pathogen interactions during a malarial (*P. cynomolgi*) infection in Rhesus macaques (*Macaca mulatta*). This model was developed using the same discrete recursive framework previously shown to be well suited for these types of problems [34].

**Fig. 1:**
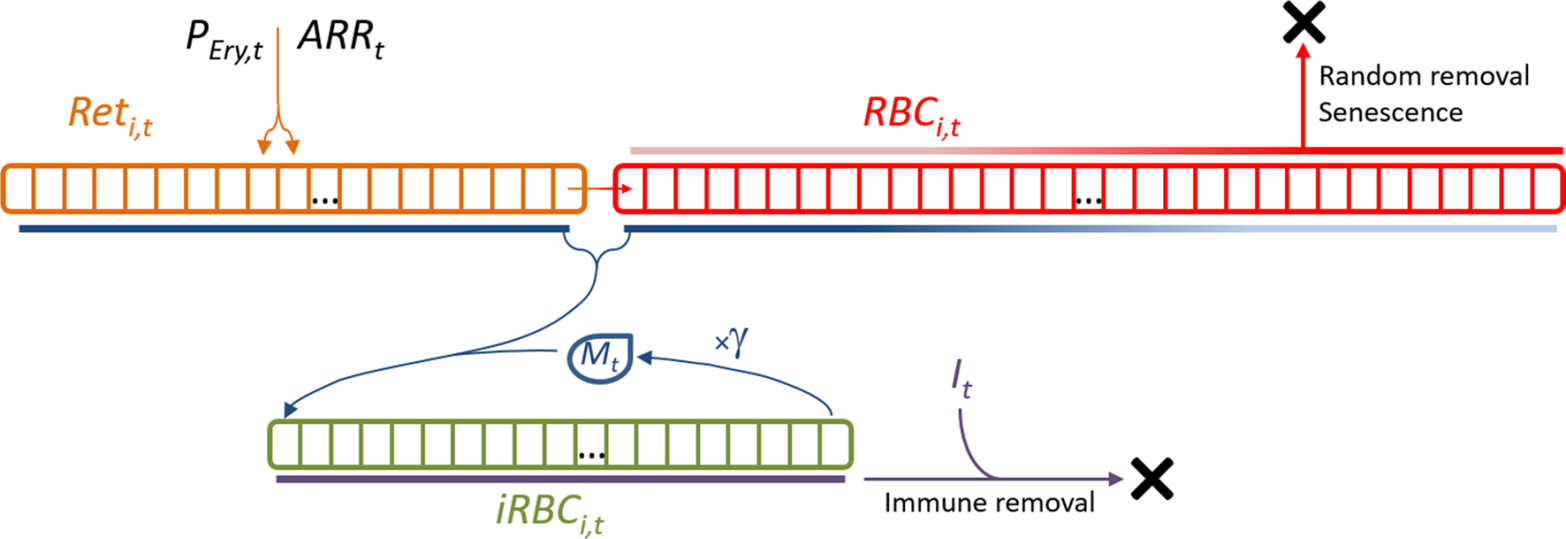
Scheme of the within-host host–pathogen interaction model. In the proposed model, reticulocytes (*Ret*) are released from the bone marrow at a rate of *P*_*t*_ cells/h/µL and with an age given by 121—*ARR*_*t*_ hours, where *ARR*_*t*_ stands for the age class into which reticulocytes are produced. All cells move from one age class to the next (right) at every hourly step. When reticulocytes reach their last age class, they mature into RBCs. In the absence of an infection, RBCs are removed at random or through senescence processes, in which older cells are more likely to be removed than young cells (depicted by the red bar over the RBC age classes). During an infection, merozoites (*M*_*t*_) will, preferentially, invade reticulocytes and young RBCs (blue bars under the reticulocyte and RBCs pools). Upon infection, reticulocytes and RBCs become infected RBCs (*iRBC*_*i,t*_), which live for 24 h, after which they burst and release *γ* new merozoites. The immune response (*I*_*t*_) removes infected RBCs in an age-independent way

In the model (Fig. 1), the erythropoietic system releases reticulocytes (*Ret*) into the bloodstream, where they remain for a certain period of time before becoming mature RBCs (*RBC*). Mature RBC may be removed by normal physiological processes, invasion by merozoites, or destruction during an infection by the so-called bystander effect. The normal physiological processes of removing RBCs include random death, a process through which about 10% of all produced cells eventually die [37], and senescence. For the latter, a hazard function was determined [38], based on data obtained through RBC biotinylation of a cohort of macaques [39] kept under the same conditions as the macaques used in the present study. The bystander effect is a process by which RBCs are removed even though they are uninfected [40, 41]; it is modelled here under the assumption that it is age-independent, as suggested previously [38].

Merozoites (*M*) invade not only mature RBCs but actually prefer reticulocytes, with a reported preference of 477:1 over RBCs [25]. The invasion of reticulocytes is assumed to be age-independent, as these cells are short-lived, lasting about 1 day in circulation. Among the mature RBCs, the parasite invasion preference is assumed to follow a decreasing exponential distribution, which is defined in such a way that the relative likelihood of infecting a young RBC, relative to a RBC 11 days older, is twice as large, $\frac{{PoI_{a} }}{{PoI_{a + 264} }} = 2$, where *PoI*_*a*_ is the probability of infecting a RBC of age *a* and *a* is the RBC age expressed in hours. The purpose of this function is to approximate the merozoite preference for younger mature RBCs and, thus, allowing the model to represent the effects of an infection on the age distribution of mature RBCs as accurately as possible. Modelling *P. cynomolgi*’s well-documented age preference for younger RBCs, as just described, does not alter the total number of RBCs removed, but allows the model simulations to produce a more realistic representation of the RBC age distribution during and after an infection.

Both, invaded RBCs and reticulocytes become infected RBCs (*iRBC*). Infected RBCs live for 48 h, which is the length of the intraerythrocytic cycle in *P. cynomolgi* [18], after which they burst and release about 16 merozoites [18]. A variable representing the immune-response (*I*) is used to control infected RBCs.

### Assumptions

The structure of the model requires very few mechanistic assumptions. In many other models, the authors assume that the formation of infected RBCs follows a mass action format [29], as it is, for instance, used in most epidemiological SIR models (i.e., $dI/dt = \beta \cdot I \cdot S - \ldots$). Here, all merozoites present at time point *t* infect RBCs or reticulocytes by time point *t *+ 1, i.e., 1 h later. Similarly, the erythropoietic output does not depend on a hypothetical function of the remaining RBCs, of the missing RBCs, or some other feature of the anaemia. Rather, a time-dependent profile (*P*_*t*_) is estimated for each macaque, given its health trajectory, which directly quantifies the RBC production at each time-point. A discrete recursive framework with age-classes is chosen, instead of an ODE, as this formulation mimics more accurately the natural physiological process of aging.

The implementation of this discrete model does require some assumptions, which are listed in Table 1.

**Table 1:** List of model assumptions

#	Description
1	The general scheme shown in Fig. 8 is assumed to represent sufficiently well the real physiological interactions between the host cells and the parasites
2	Reticulocytes are produced and released from the bone marrow at a rate of *P*_*t*_ and with a remaining maturation time of 121 *ARR*_*t*_, at the end of which they become mature RBCs
2.1	Reticulocytes are not subject to random or senescent death as they become mature RBCs within one to 2 days after entering circulation
2.2	Reticulocytes may be infected by the parasite
3	Mature RBCs are, theoretically, allowed to live for up to 160 days
3.1	Random death removes 10% of all produced RBCs
3.2	Senescent death follows a hazard function that causes RBCs to have a mean lifespan of 98 days
3.3	Mature RBCs can be lost due to invasion by a parasite
3.4	Mature RBCs may be lost due to the bystander effect
4	Free merozoites live only for 1 h, during which time they infect reticulocytes and RBCs
4.1	Merozoites have a reported 477.2:1 preference for reticulocytes over RBCs
4.2	Among mature RBCs, merozoites have an age preference that leads a younger RBC to be two times more likely to be infected than a RBC 11 days older
5	Infected RBCs live for 2 days and then burst, releasing a new brood of merozoites
5.1	Infected RBCs are removed by the immune response

### Variables

The model contains five dependent variables: *Ret*_*i,t*_, *RBC*_*i,t*_, *iRBC*_*i,t*_, *rRBC* and, *M*. The definitions of all model variables are given in Table 2.

**Table 2:** Definition of all variables. The first five variables (*Ret*_*i,t*_, *RBC*_*i,t*_, *iRBC*_*i,t*_, *rRBC* and, *M*) are the key dependent variables of the model for which time recursions are defined

Variable	Description
*Ret*_*i,t*_	Number of circulating reticulocytes of age *i* at time *t*
*RBC*_*i,t*_	Number of mature red blood cells of age *i* at time *t*
*iRBC*_*i,t*_	Number of infected red blood cells of age *i* at time *t*
*rRBC*_*t*_	Number of RBCs that will die due to random death
*M*_*t*_	Number of free merozoites at time *t*
*P*_*t*_	Hourly production and release of reticulocytes from bone marrow into the blood-stream at time *t*
*ARR*_*t*_	Age-class into which reticulocytes are released
*iARR*_*t*_	Integer part of *ARR*_*t*_
*dARR*_*t*_	Decimal part of *ARR*_*t*_
*SfRet*_*i,t*_	Fraction of reticulocytes in age class *i* at time point *t* that survive to time point *t *+ 1
*SfRBC*_*i,t*_	Fraction of RBCs in age class *i* at time-point *t* that survive to time point *t *+ 1
*SfiRBC*_*i,t*_	Fraction of infected RBCs in age class *i* at time point *t* that survive to time point *t *+ 1
*ToI*	Time point at which an infection starts
*NoI*	Number of infected RBCs that start an infection at time point *ToI*
*RDt*	Number of RBCs removed at time point *t* due to random death
*hf*_*i*_	Hazard function for RBCs
*RBCInv*_*i,t*_	Fraction of RBCs in age class *i* at time point *t* lost to merozoite invasion
*RetInv*_*i,t*_	Fraction of reticulocytes in age class *i* at time point *t* lost to merozoite invasion
*UR*_*t*_	Number of RBCs removed by bystander effect
*TRBC*_*t*_	Total number of RBCs at time *t*
*TRet*_*t*_	Total number of reticulocytes at time *t*
*Ret*_*Pref*_	Reticulocyte preference over mature RBCs
*RBCAPf*_*i*_	Merozoite-RBC age preference function
*PiRet*	Percentage that a merozoite will infect a reticulocyte
*NiRet*	Number of reticulocytes to be infected
*PiRBC*_*i*_	Probability that a merozoite will infect an RBC of age *i*
*MerLeft*	Number of merozoites left after accounting for invasion of reticulocytes
*NiRBC*_*f*_	Number of RBCs of age class *f* to be invaded by a merozoite

### Equations

The within-host host–pathogen interaction model is set up as a discrete recursive system and solved with a 1-h time step. The next paragraphs describe each compartment and the formulation of all associated processes.

#### Reticulocyte compartment

In primates, reticulocytes are produced in the bone marrow and released into circulation before maturing into RBCs. The common maturation time in circulation for human reticulocytes is about 24 h, but can change in response to anaemia [42]. To account for this variable maturation time, the reticulocyte pool (*Ret*_*i,t*_) is defined as having a total of 120 age classes, which corresponds to a maximum of 5 days or 120 h (Eq. 1). Reticulocytes are produced at a rate of *P*_*t*_ into age class *ARR*_*t*_ (Fig. 1), and then allowed to age until they reach their last age class, 120, at which point the reticulocytes become mature RBCs. In order for the model to respond in a quasi-continuous fashion to *ARR*_*t*_, newly released reticulocytes are distributed among the two age classes *int*(*ARR*_*t*_) and *int*(*ARR*_*t*_) + 1 based on the decimal part of *ARR*_*t*_ (Eqs. 1, 3). *P. cynomolgi*, unlike *P. coatneyi*, has the ability to invade reticulocytes and does so with a 477:1 preference over RBCs, according to [25]. Therefore, reticulocyte removal was introduced using *SfRet*_*i,t*_ (Eqs. 1, 13, 24). Modelled this way, a normal 24-h maturation time in circulation for reticulocytes is modelled by these cells being released into age class *ARR*_*t*_ = 121 − 24 = 97, which ensures that 24 h later they become mature RBCs. Thus, the equations governing reticulocytes are given as follows:

1 $$\left\{ \begin{array}{ll} Ret_{i,t + 1} = 0 & \quad i = 1 \\ Ret_{i,t + 1} = Ret_{i - 1,t} \cdot SfRet_{i - 1,t} & \quad i \in \{ 2, \ldots ,iARR_{t} - 1\} \\ Ret_{i,t + 1} = Ret_{i - 1,t} \cdot SfRet_{i - 1,t} + P_{t} \cdot dARR_{t} & \quad i = iARR_{t} + 1 \\ Ret_{i,t + 1} = Ret_{i - 1,t} \cdot SfRet_{i - 1,t} + P_{t} \cdot dARR_{t} & \quad i \in \{ iARR_{t} + 2, \ldots ,120\} \\ \end{array} \right.$$

2 $$Ret_{i,0} = \left\{ {\begin{array}{*{20}ll} 0 &\quad {i \in \left\{ {1, \ldots ,iARR_{0} - 1} \right\}} \\ {P_{0} \cdot \left( {1 - dARR_{0} } \right)} &\quad {i = iARR_{0} } \\ {P_{0} } &\quad {i \in \left\{ {iARR_{0} + 1, \ldots ,120} \right\}} \\ \end{array} } \right.$$

Here, *iARR*_*t*_ and *dARR*_*t*_ are the integer and decimal parts of *ARR*_*t*_ (age class into which reticulocytes are released, Table 2):

3 $$iARR_{t} = int\left( {ARR_{t} } \right)\quad {\text{and}}\quad dARR_{t} = ARR_{t} - iARR_{t}$$

#### RBC compartment

The RBC compartment (*RBC*_*i,t*_) has 3840 (= 160 × 24) age classes (Eq. 4), which allows for a maximum lifespan of 160 days. Although RBCs live on average only for 98 days in Rhesus macaques [38], significant numbers survive to about 120 days and sometimes longer [38]. The remaining 40 age classes ensure that even under conditions where large numbers of RBCs are produced in a short period of time, RBCs never “artificially” disappear due to a lack of age classes. In most simulations, the oldest age classes are empty. RBCs are produced from reticulocytes by maturation, and removal occurs by two sets of processes: physiological and pathophysiological. The two normal, physiological processes are random loss and senescence. Random loss of RBCs is modelled under the assumption that 10% of all RBCs produced will die by this process; this mechanism is modelled with the auxiliary variable *rRBC*_*t*_ [37, 38]. Senescent death is governed by a power-law hazard function, which was previously parameterized for Rhesus macaques [38]. The pathophysiological processes are merozoite invasion and RBC removal by bystander effect. Merozoites are released at the end of the intraerythrocytic developmental cycle and within 1 h re-infect RBCs and reticulocytes (Fig. 1). The bystander effect is an ill-characterized process that is known to occur during some malarial infections and leads to additional, sometimes substantial, losses of RBCs. Fonseca et al. [38] recently suggested that the bystander mechanism removes RBCs in an age-independent manner, and that it does not seem to occur due to an increased rate of RBC senescence. Consequently, the bystander effect is modelled as an age-independent process, namely

4 $$RBC_{i,t + 1} = RBC_{i - 1,t} \cdot SfRBC_{i - 1,t} , \qquad i \in \left\{ {2,3, \ldots ,3840} \right\}$$

5 $$RBC_{1,t + 1} = Ret_{120,t} \cdot Sfret_{120,t} .$$

The initial state *RBC*_*i,*0_ may be analytically deduced, but this deduction is complex. Instead, it was numerically calculated by starting without any RBCs (*RBC*_*i,*0_ = 0) and running the model absent of infection or loss of RBCs by bystander effect for over 1500 days, when it had long reached the steady state. Under these conditions, the model is asymptotically stable at the initial state. This final RBC age distribution (*RBC*_*i,*end_) is saved and used as the initial state for all subsequent simulations.

#### Merozoite compartment

Merozoites are produced in infected RBCs and released upon completion of the intra-erythrocytic developmental cycle (Fig. 1). Although *P. cynomolgi* is known to produce an average of 16 merozoites, with a range of 14–20 [18], some of the macaques analysed here exhibited higher rates. In view of this fact, this parameter was individually fitted for each parasitaemia peak in each macaque assuming the growth of the parasite population follows an exponential function and calculating the multiplication rate as the increase in numbers (*γ*) during 48 h. Therefore, the number of merozoites released will be *γ* times the number of surviving infected RBCs in their last age class.

6 $$M_{t + 1} = \gamma \cdot iRBC_{48,t} \cdot SfiRBC_{48,t}$$

7 $$M_{t = 0} = 0$$

Note that *M*_*t*+1_ does not depend on *M*_*t*_, because the free merozoites present at time *t* infect RBCs within 1 h (Eq. 9) since their appearance in blood. The merozoites that will be present at time *t *+ 1 are those released from infected RBCs that finished their intra-erythrocytic developmental cycle at time *t* and were not removed by the immune-response (Eq. 6).

#### Compartment of infected RBCs

Infected RBCs (*iRBC*_*i,t*_) are produced from merozoite invasion of RBCs or reticulocytes and destroyed by the immune system (*I*_*t*_, Fig. 1). All infected RBCs that survive for the entire intra-erythrocytic developmental cycle of 48 h are assumed to burst and release *γ* merozoites each. It is furthermore assumed that all released merozoites are infectious. Additionally, since *P. cynomolgi* has a 2-day intra-erythrocytic cycle, this compartment is modelled with 48 age classes.

8 $$iRBC_{i,t + 1} = iRBC_{i - 1,t} \cdot SfiRBC_{i - 1,t} , \quad i \in \left\{ {2,3, \ldots ,48} \right\}$$

9 $$iRBC_{1,t + 1} = M_{t}$$

10 $$iRBC_{i,0} = 0, \quad i \in \left\{ {1,2, \ldots ,48} \right\}$$

Infections are initiated in the model (Eq. 11) by stating how many infected RBCs (*NoI*) exist at the time the infection starts (*ToI*), close to the time the infection becomes patent. Here, it is assumed that the infection has a uniform age-distribution at the time of patency; i.e., all parasite life stages are equally present at any time point as shown by the experimental data. Therefore:

11 $$iRBC_{i,t = TOI} = \frac{NoI}{48}, \quad i \in \left\{ {1,2, \ldots ,48} \right\}$$

#### Destruction of RBCs and reticulocytes

While reticulocytes are assumed to be destroyed only by merozoite invasion, RBCs are assumed to be removed by random death, senescence, merozoite invasion or bystander effect (Fig. 1). Thus, the fractions of surviving RBCs and reticulocytes are:

12 $$SfRBC_{i,t} = \left( {1 - \frac{{RD_{t} }}{{TRBC_{t} }} - hf_{i} - RBCInv_{i,t} - \frac{{UR_{t} }}{{TRBC_{t} }}} \right), \quad i \in \left\{ {1,2, \ldots ,3840} \right\}$$

13 $$SfRet_{i,t} = \left( {1 - RetInv_{i,t} } \right), \quad i \in \left\{ {1,2, \ldots ,120} \right\}$$

where *RD*_*t*_ is the number of RBCs that are removed by random destruction, *hf*_*i*_ is the RBC hazard function that determines the fraction of RBCs lost due to senescence, *RBCInv*_*i,t*_ is the fraction of RBCs lost to invasion, *UR*_*t*_ is the number of RBCs removed by the bystander effect, *RetInv*_*i,t*_ is the fraction of reticulocytes lost to invasion, and

14 $$TRBC_{t} = \mathop \sum \limits_{i = 1}^{3840} RBC_{i,t} .$$

Random death is modelled as:

15 $$rRBC_{t + 1} = rRBC_{t} + 0.1 \cdot P_{t} - \frac{{rRBC_{t} }}{800}$$

16 $$RD_{t} = \frac{{rRBC_{t} }}{800}$$

17 $$rRBC_{0} = 80 \cdot P_{t}$$

where *RD*_*t*_ is given by the number of RBCs lost through Eq. (15). This removal process is characterized by a first-order rate of (800 h)^−1^, which was approximated from the corresponding rate in humans (1024^−1^) [37, 38] by rescaling to ≈ 4*/*5, given the ratio of RBC lifespans in macaques and humans of 100 and 120 days, respectively.

The hazard function used here was obtained elsewhere [38] as

18 $$hf_{i} = 8.488 \cdot 10^{ - 45} \cdot i^{12.25} ,\quad i \in \left\{ {1, 2, \ldots , 3840} \right\}.$$

The hazard function is defined for the 3840 age classes of RBCs, which corresponds to 160 days and thus the maximum theoretical lifespan. In practice, the average RBC is removed at an age of 98 days. RBC and reticulocyte destruction by merozoite invasion was implemented as described below and is only executed if merozoites are present. Once the presence of merozoites has been ascertained, the algorithm checks if there are more RBCs than merozoites (*T RBC*_*t*_+ *T Ret*_*t*_>* M*_*t*_). If merozoites outnumber RBCs, the simulation is stopped due a lack of RBCs, which implies that the host has died. The total number of reticulocytes is given by:

19 $$TRet_{t} = \mathop \sum \limits_{i = 1}^{120} Ret_{i,t} .$$

Taking into consideration the reticulocyte-to-RBC preference (*Ret*_*Pref*_) [25] and the RBC age preference (*RBCAPf)*, given by

20 $$Ret_{Pref} = 477.2,$$

21 $$RBCAPf_{i} = e^{{ - \frac{10i}{3840}}} , \quad i \in \left\{ {1,2, \ldots ,3840} \right\},$$

the percentage of merozoites infecting reticulocytes is calculated as:

22 $$PiRet = \frac{{Ret_{Pref} \cdot TRet_{t} }}{{Ret_{Pref} \cdot TRet_{t} + TRBC_{t} }}.$$

Thus, the number of reticulocytes being infected is:

23 $$NiRet = \left\{ {\begin{array}{*{20}l} {M_{t} \cdot PiRet,} & {if \, M_{t} \cdot PiRet < TRet_{t} } \\ {TRet_{t} ,} & {if \, M_{t} \cdot PiRet \ge TRet_{t} } \\ \end{array} } \right.$$

Equation (23) ensures that even at high reticulocyte/RBC preference rates and low RBC numbers, no more than the actually existing reticulocytes are removed. The fraction of reticulocytes lost to invasion (*RetInv*_*i,t*_) is then given by:

24 $$RetInv_{i,t} = \frac{NiRet}{{TRet_{t} }},\quad i \in \left\{ {1,2, \ldots ,120} \right\},$$

which guarantees that the same proportion of reticulocytes is removed from every age class. In other words, the invasion of reticulocytes is assumed to be age-independent. At this point, there are still *M*_*t*_− *N iRET* merozoites available to infect RBCs, and the removal of these RBCs follows the age-preference function (*RBCAPf*_*i*_). This removal is accomplished with the auxiliary function described in supplemental materials (Additional file 1).

### Removal of infected RBCs by the immune system

In order to account for the immune system’s ability to control the infection, an immune response function is introduced, which leads to the removal of infected RBCs:

25 $$SfiRBC_{i,t} = \left( {1 - I_{t} } \right),\quad i \in \left\{ {1, 2, \ldots , 48} \right\}.$$

### Modelling the healthy state

A healthy macaque is characterized by a steady state in terms reticulocytes and RBCs. For this state, only two time-invariant parameters need to be determined, namely, the erythropoietic output, which corresponds to RBC production and release rate, *P0*_*MacaqueX*_, and the reticulocyte maturation time in circulation, which is determined as the age of that which reticulocytes are released from the bone marrow; it is given as *ARR*0_*MacaqueX*_. The steady state is achieved by specifying that there is no infection, no immune response, and no bystander effect.

26 $$\left\{ {\begin{array}{*{20}l} {P_{t} = P0_{MacaqueX} } \\ {ARR_{t} = ARR0_{MacaqueX} } \\ {M_{t} = 0,\quad \forall t > 0} \\ {NoI = 0} \\ {ToI = + {\kern 1pt} \infty } \\ {UR_{t} = 0, \quad \forall t > 0} \\ {I_{t} = 0, \quad \forall t > 0} \\ \end{array} } \right.$$

The parameters *P*0_*MacaqueX*_ and *ARR*0_*MacaqueX*_ were obtained by optimization of the model, as defined in Eq. (26), against reticulocyte and RBC data obtained either for healthy macaques (E13 cohort, see below) or for infected macaques (E04 and E03 cohorts, see below). In the case of infected macaques, only the first 5- to 7 days of data are used, as long as these show no changes. The optimizations were performed in MatLab using the (*fminsearch*) algorithm to solve the nonlinear least-square problem.

### Modelling an infected blood profile of a macaque

The modelling of an infected macaque’s profile starts by calculating its healthy haematological parameters, as shown above for a non-infected macaque. Next, the infected RBC profile is modelled by optimization of the *NoI* and *ToI* parameters, which cause the blood infection to appear at a certain point in time (*ToI*) and, at a certain level (*NoI*). The infected RBC profile is fitted by optimization of the immune response (*I*_*t*_).

The time-dependent characterization of the infection is accomplished by adjusting the remaining physiological responses, namely the erythropoietic output, *P*_*t*_; reticulocyte maturation time, *ARR*_*t*_; and bystander effect, *UR*_*t*_. In order to transform the determination of a temporal profile into a standard parametric estimation problem, these functions are modelled as sums of peak functions (Eqs. 27–30)

27 $$P_{t} = P0_{MacaqueX} \cdot \left( {1 + \mathop \sum \limits_{i} f\left( {t|\alpha_{1,i} ,\beta_{1,i} ,\gamma_{1,i} ,\delta_{1,i} ,\varepsilon_{1,i} } \right)} \right)$$

28 $$ARR_{t} = ARR0_{MacaqueX} + \mathop \sum \limits_{i} f\left( {t|\alpha_{2,i} ,\beta_{2,i} ,\gamma_{2,i} ,\delta_{2,i} ,\varepsilon_{2,i} } \right)$$

29 $$UR_{t} = \mathop \sum \limits_{i} f\left( {t|\alpha_{3,i} ,\beta_{3,i} ,\gamma_{3,i} ,\delta_{3,i} ,\varepsilon_{3,i} } \right)$$

30 $$I_{t} = \mathop \sum \limits_{i} f\left( {t|\alpha_{4,i} ,\beta_{4,i} ,\gamma_{4,i} ,\delta_{4,i} ,\varepsilon_{4,i} } \right)$$

where the following peak function is used due to its flexibility (Eq. 31) in generating symmetrical and asymmetrical peaks and plateaus:

31 $$f\left( {t|\alpha ,\beta ,\gamma ,\delta ,\varepsilon } \right) = \alpha \cdot \frac{1}{{1 + \beta^{\gamma - t} }} \cdot \left( {1 - \frac{1}{{1 + \delta^{\varepsilon - t} }}} \right), \quad \varepsilon > \gamma .$$

This function (Eq. 31) results from the juxtaposition of two logistic functions, where *α* represents the top of the peak or plateau; *β* and *δ* allow the modulation of the rate of ascent and descent, respectively; and *γ* and ε define the points in time where half of the ascent and of the descent occurs, respectively.

All responses (Eqs. 27–30) are modelled with a sum of a variable number of peak functions. In this way the characterization of the response profile of a macaque is converted into a parameter estimation problem, which was implemented in MatLab using a Levenberg–Marquardt algorithm (*lsqnonlin*) that solves the nonlinear least-squares problem.

### Experimental datasets

Three datasets are used in this work; they have been denoted as ‘E04’, ‘E03’, and ‘E13’ in the malaria host–pathogen interaction consortium (MaHPIC). ‘E04’ is the main dataset, which is used throughout this work, whereas ‘E03’ and ‘E13’ are used only to provide additional data for the characterization of the maturation time of reticulocytes in circulation and the reticulocyte production rate. A summary of each of these cohorts is provided below; more detailed descriptions and data are also publicly available [35].

#### E04 cohort

The ‘E04’ experiment was performed to study *P. cynomolgi* infections in Rhesus macaques (*Macaca mulatta*). A detailed clinical analysis of the results of this experiment has been published elsewhere [17]. Briefly, five malaria-naïve male macaques, with designations RFa14, RIc14, RMe14, RSb14, RFv13, born and raised at the Yerkes National Primate Research Center, were infected with *P. cynomolgi* B strain through an intravenous inoculation of 2000 freshly-isolated sporozoites per animal. The animals were followed for 100 days, during which ear-prick blood samples were collected daily and analysed for complete blood counts (CBC) and parasitaemia counts.

Four macaques of the E04 cohort (RFa14, RIc14, RMe14, RSb14) survived the 100-day infection study [17]. Macaque RFv13 had to be euthanized due to acute kidney failure and was excluded from this modelling analysis, as it was never able to mount an effective response to the infection. Two of the remaining macaques, RFa14 and RMe14, had a severe clinical outcome and received sub-curative anti-malaria treatments. RMe14 was also provided with a blood transfusion as a precautionary measure given that its haemoglobin levels approached 6 g/dl.

#### E13 cohort

The ‘E13’ experiment was performed as a control for other studies. In this experiment, healthy macaques were treated in the same way as other cohorts, but never infected with malaria. The E13 cohort consisted of five malaria-naïve male Rhesus macaques which were followed for 100 days, without ever being exposed to malaria, and were treated with pyrimethamine on days 26, 57, 58, 59, 95, 96 and 97. Pyrimethamine treatment had no effect on blood cell counts [43], and therefore can be considered a true control experiment.

#### E03 cohort

The design of experiment ‘E03’ was very similar to the design of ‘E04’. The target was, however, to study the effects of a different malaria parasite species, *P. coatneyi*, on Rhesus macaques. Specifically, five malaria-naïve male Rhesus macaques were infected with *P. coatneyi* sporozoites and followed for 100 days during which many samples were taken for different measurements, including daily CBC and parasitaemia levels [43]. Here, only the first 7 days of RBC and reticulocyte measurements are used.

### Experimental validation of the reticulocyte maturation time in circulation

#### In vitro macaque blood cultures

Venous blood from healthy Rhesus macaques was collected in EDTA. A buffy coat preparation was then performed by centrifuging each sample at 200×*g* for 10 min. After centrifugation, the buffy coat was removed and discarded. The remaining red blood cell pellet was washed four times in RPMI by centrifugation at 400×*g* for 10 min. After washing, the RBC pellet was resuspended in complete RPMI supplemented with l-glutamine, 0.25% sodium bicarbonate, 50 µg/mL hypoxanthine, 7.2 mg/mL HEPES, 2 mg/mL glucose, and 10–20% Human AB + serum to 10% haematocrit. The 10% haematocrit solution was aliquoted into close-cap culture flasks and incubated at 37 °C under blood gas conditions (5%:5%:90%; O_2_:CO_2_:N_2_). Samples were taken at 0, 4, 20, 25.5, and 48 h after incubation to enumerate reticulocytes by flow cytometry and by new methylene blue staining.

#### Monitoring reticulocyte maturation by flow cytometry

Twenty microlitres of the RBC culture were taken from each culture flask and washed in PBS by centrifugation at 800×*g* for 7 min. After washing, the supernatant was aspirated and discarded. The remaining RBC pellet was then resuspended in a cocktail of fluorescently-conjugated antibodies (Clone D058-1283; Fluorochrome APC-Cy7). After resuspension, the antibodies were incubated for 15 min in the dark at room temperature. The samples were then washed in PBS by centrifugation at 800×*g* for 7 min. The supernatant was discarded, and the remaining RBC pellet was resuspended for flow cytometry analysis. Data were acquired immediately without fixation using a BD LSR Fortessa flow cytometer. Raw FCS files were imported into FlowJo and compensated followed by importing the compensated files into the Cytobank platform for analysis.

#### Reticulocyte enumeration by new methylene blue staining

Ten microlitres of the RBC culture were taken from each culture flask and incubated with ten microlitres of new methylene blue for 20 min at 37 °C. After incubation, a thin blood smear was made and allowed to dry prior to the determination of the number of reticulocytes in the sample. For each sample, the number of reticulocytes out of 1000 RBCs was determined, and the percentage of reticulocytes in each sample was calculated for analysis.

## Results

### Host-parasite interaction model

**Fig. 2:**
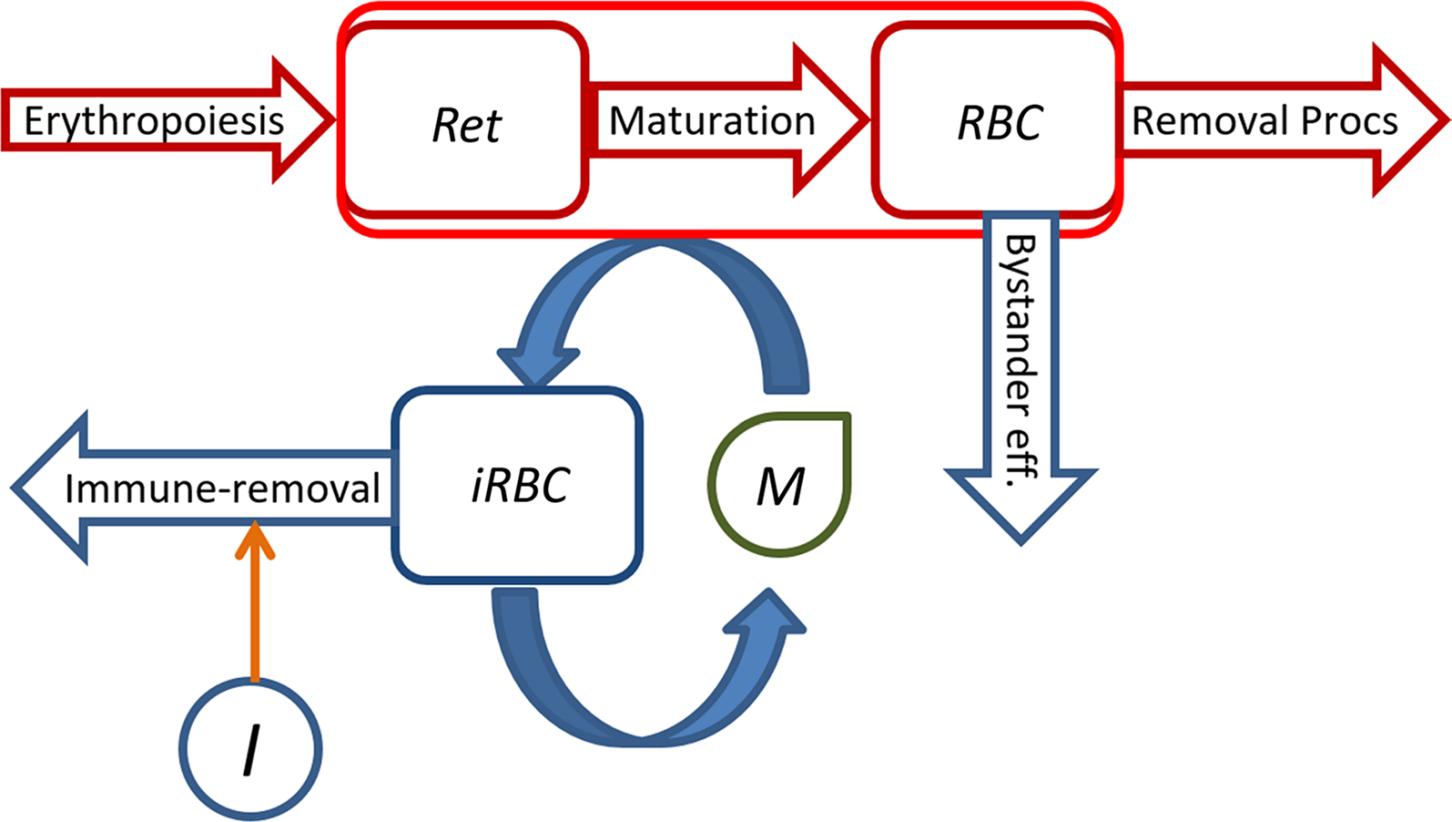
Simplified diagram of the model. In the absence of an infection, reticulocytes (*Ret*) are produced through *erythropoiesis* and mature into RBCs (*RBC*). These are prone to be removed by the physiological removal processes (*Removal Procs*). In the presence of an infection (processes depicted in blue), merozoites (*M*) invade both reticulocytes and RBCs, thereby generating infected RBCs (*iRBC*). At the end of the parasites’ intraerythrocytic development cycle, the infected RBCs burst and release a new brood of merozoites. Up-regulation of the immune-response (*I*) leads to removal of infected RBCs and ideally to control of the parasitaemia. The presence of an infection may also cause loss of RBCs by other means besides parasite invasion (*Bystander eff.*)

The proposed model of the malaria blood-stage dynamics uses a discrete recursive framework with a 1-h time-step and an age-structure for reticulocytes, RBCs and infected RBCs [34]. A simplified diagram of the model structure is shown in Fig. 2. The model assumes that reticulocytes are released from the bone marrow at a given rate and age (Eq. 1). At the end of their maturation time, the reticulocytes become mature RBCs. These RBCs have an average lifespan of 98 days in Rhesus macaques [38] and, under normal conditions, are removed due to either senescence or random death. These processes were mathematically characterized elsewhere [38] and are reused here. Upon infection of the blood, the *Plasmodium cynomolgi* merozoites are able to invade reticulocytes and mature RBCs. *P. cynomolgi* within infected RBCs complete their intra-erythrocytic developmental cycle in 2 days, after which the RBCs burst and release a new brood of merozoites. During the infection, the increased level of destruction of RBCs is not only attributable to invasion by merozoites but may also be due to a significant bystander effect, which is included in the model. The infection may be controlled either by the host through an up-regulation of the immune response which causes the removal of infected RBCs, or by anti-malarial treatment regimens.

### Reticulocyte maturation time in Rhesus macaques

In control animals, RBC and reticulocyte numbers stay essentially constant, absent of any insult [43]. Therefore, model optimization against control macaque data allows the inference of the haematological parameters for reticulocyte release rate and maturation time. In the case of infected macaques, it was assumed that the haematological steady state, observed between the pre-infection period and the first 5 days post-infection, represents reasonably well the non-infected baseline state of each macaque. Using this procedure, the reticulocyte release rate and maturation time were calculated for each macaque in three separate cohorts: E04 [17] (the main cohort modelled in this paper), E03 (Cordy et al. pers. comm.) and E13 [43]. The data for these cohorts are publicly available [35, 43].

**Fig. 3:**
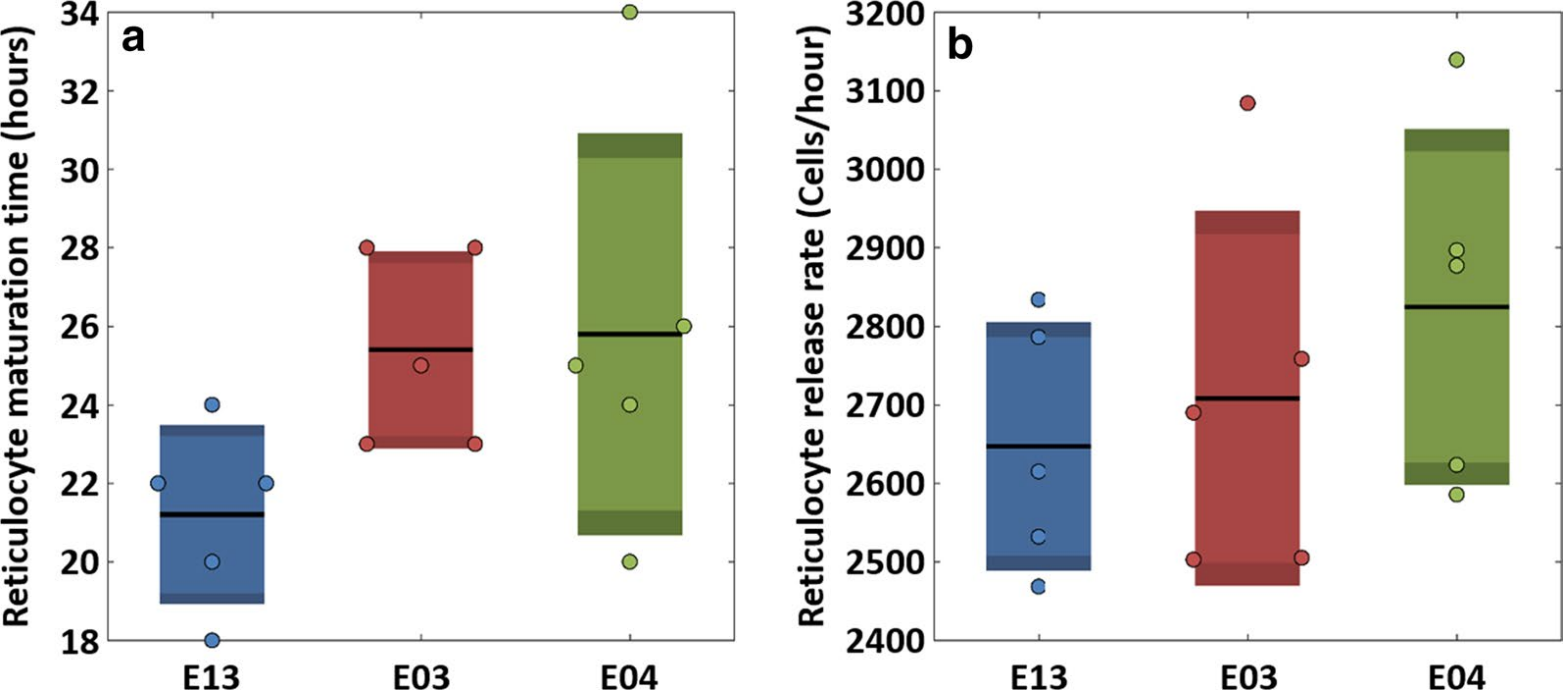
Reticulocyte maturation time and release rate for healthy Rhesus macaques. **a** The distributions of reticulocyte maturation times obtained for three five-monkey cohorts (E13, E03 and E04), while **b** shows the distributions of reticulocyte release rates for the same cohorts. Dots represent values obtained for each monkey; black line: mean; lighter coloured bar: 95% standard error of the mean; darker coloured bar: standard deviation in each cohort

Analysis of the results obtained for these three cohorts (Fig. 3) shows only modest inter-cohort differences, and the overall averages for the reticulocyte maturation time in circulation and the reticulocyte release rate are 24 ± 5 h and 2727 ± 209 cells/h/µL, respectively.

**Fig. 4:**
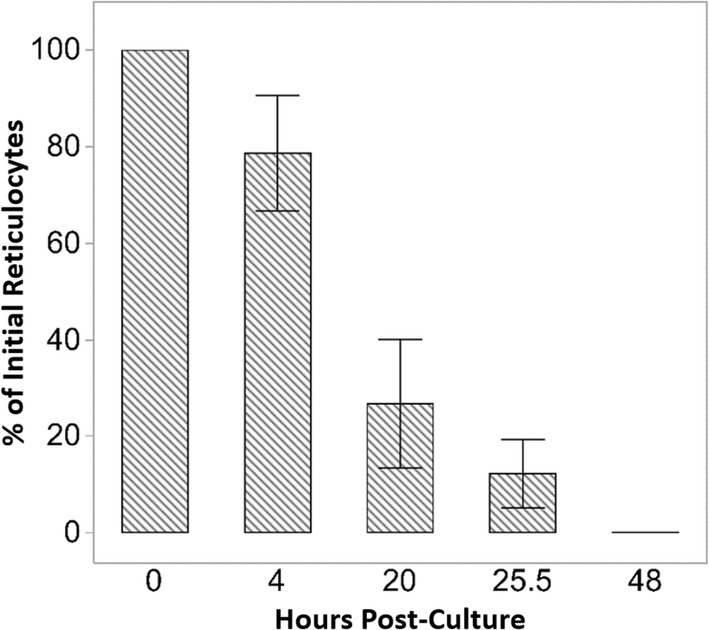
Lifespan of Rhesus macaque reticulocytes in vitro. Loss of red blood cells staining positive for RNA via a new methylene blue stain (i.e., reticulocytes) over a 48 h period for 6 Rhesus macaques. Results are from two independent experiments. Error bars = SEM

To validate the inferred in vivo reticulocyte maturation time in circulation, freshly drawn RBCs from two different healthy macaques were incubated in vitro, and reticulocytes followed over time (Fig. 4). The reticulocytes were assessed by measuring RBCs staining positive for RNA (Fig. 4). Using the former method, fewer than 20% of the reticulocytes were found after 25 h of incubation in vitro (Fig. 4), which is similar to what was determined in vivo (Fig. 3).

### Characterization of responses to malarial infections

The optimization of the model towards reproducing the experimental blood profile of a given macaque leads to inferences regarding the temporal host response to the infection. This characterization includes the profile of the reticulocyte release rate (or erythropoietic output); the reticulocyte maturation time in circulation; the RBC removal due to bystander effect, parasitization, senescence, and random processes; and the immune response.

### Modelling RFa14

**Fig. 5:**
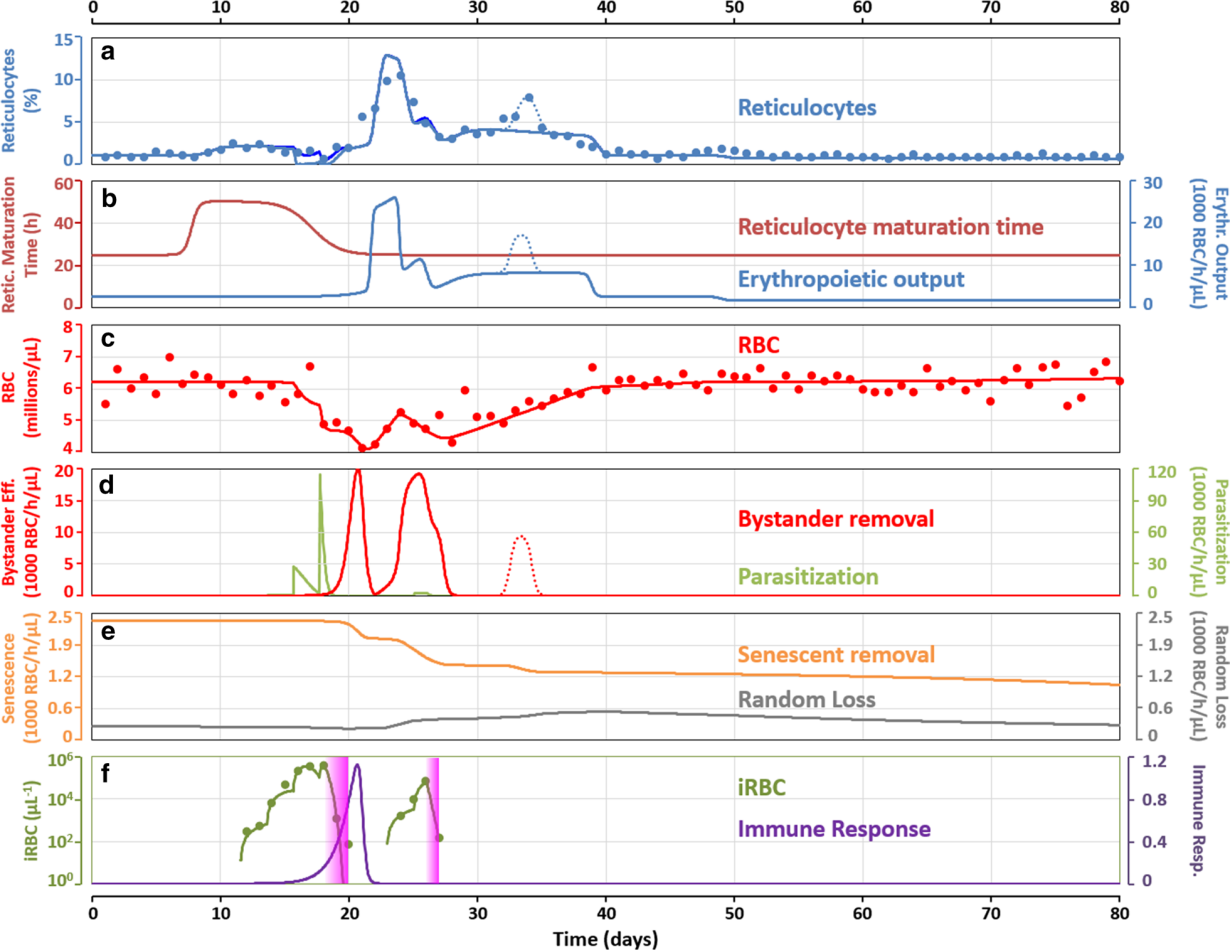
Characterization of the infection in RFa14. **a**, **c**, **f** The experimentally obtained data for reticulocytes, RBCs, and infected RBCs with the corresponding model fits. **a** Also exhibits the result of an alternative model, in dark blue solid line, which is obtained for a reticulocyte-RBC preference ratio of 15:1 and fits the data better than the 477:1 ratio shown in lighter solid blue. **b** Time courses for the reticulocyte maturation time and erythropoietic output. **d** Time courses of the numbers of cells being lost due to the bystander effect and by parasitization. **e** Time courses of the numbers of cells being lost by senescent removal and random loss. **f** Experimental data for infected RBCs (green dots), model fit to the infected RBCs (green line), immune response time course (purple line), and sub-curative and curative treatment windows (pink boxes; the left side of the boxes depicts the experimental point-of-treatment, while the window width depicts the modelled time course of progressive cell removal, until the right side of the box is reached, where the model assumes that all infected RBCs have been killed by the treatment). Results of a second alternative model are shown in **a**, **b**, **d** as dotted lines. This alternative model fit is obtained assuming an exactly equal increase in RBC production and loss due to bystander effect between Days 33 and 35. Although unlikely, this hypothetical setting does lead to an increase in reticulocytes without any change in RBC numbers

Applying the model to the data obtained for RFa14 (Fig. 5a, c, f) permits the inference of this macaque’s infection response profile (Fig. 5b, d, e, f). During the first 7 days, this macaque presented with a healthy phenotype (Fig. 5a, c, f), which allowed an assessment of its baseline values. The reticulocyte maturation time in circulation was determined to be 25 h, and the erythropoietic output was 2623 RBCs/h/µL (Fig. 5b). During this same interval, RBCs were mainly removed due to senescence (2361 RBCs/h/µL, Fig. 5e), and considerably fewer cells were lost through random processes (262 RBCs/h/µL).

At days 8 and 9, the maturation time of reticulocytes started to increase, suggesting that these cells started to be released from the bone marrow at a younger stage and giving rise to an increased percentage of reticulocytes (Fig. 5a), without a corresponding change in RBC numbers (Fig. 5c). This trend slowly became stronger, and on day 11, reticulocytes were being released 25 h younger than normal. Upon release, these cells subsequently spent a total of 50 h in circulation before maturating into RBCs, except if they happened to be infected before completing their maturation.

The observed growth rate of the parasite in this macaque, expressed as the number of merozoites released per infected RBC in a 48 h cycle, was 54.4 and 40.6 for the first and second parasitaemia peaks, respectively (ranges 11–19 and 23–26 days). These values are much larger than what is expected for *P. cynomolgi*, as this parasite is known to release, on average, 16 merozoites per infected RBC, with a range of 14–20. While at first counterintuitive, these results have been shown to be suggestive of concealment of the infected RBCs in tissues, away from the peripheral circulation [44]. In the present model, the assumption of a concealed population of infected RBCs was not used. The model assumes a rate of parasite growth equal to the observed rate. In this way, the observed growth kinetics is modelled, but no assumption or information on the magnitude and kinetics of the concealed population are required.

Infection patency occurred at day 12, and shortly thereafter, RBC death by parasitization became significant, causing the RBC and reticulocyte titers to start tumbling. Accounting for *P. cynomolgi*’s reported 477:1 preference for reticulocytes over mature RBCs [25] leads the model to predict a complete removal of reticulocytes at peak parasitaemia (Days 16–18, Fig. 5a). This result is in stark contrast to the experimental data, which indicate only a modest drop in reticulocytes (Fig. 5a). To explore this discrepancy further, alternate levels of the reticulocyte/RBC preference were tested, and a ratio of 15:1 emerged to fit the data best. The model predictions with this alternate parameter value are shown in dark blue (Fig. 5a). This new parameter value does not change any other model outputs, since the same numbers of RBCs are still being removed. Apart from the time period between days 16 and 18, the effects of this adjusted parameter value are only noticeable at day 26. During peak parasitaemia, the release of younger reticulocytes subsides, and the reticulocyte maturation time returns to 25 h.

Interestingly, the model predicts that the bystander effect increases after the sub-curative treatment and peaks just before Day 21. By Day 22, the bystander effect subsides, and RBC production increases (Fig. 5b). This peak of RBC production is responsible for the peak of reticulocytes between Days 22–25 (Fig. 5a) and for the increase in RBC numbers between Days 22 and 24 (Fig. 5c). To reproduce these two observations, the model predicts an erythropoietic output increase from its baseline value of 2623 to 26,080 RBCs/h/µL. This tenfold jump is able to replenish approximately half of the RBCs lost up until Day 20. Between Days 23 and 27, a new smaller peak of parasitaemia develops. This peak is accompanied by RBC losses that are mainly due to the bystander effect (Fig. 5d), given that the relatively low levels of parasites (Fig. 5f) cannot explain the numbers of RBCs lost (Fig. 5b).

At Day 26, this macaque received curative treatment, and the last day of recorded parasitaemia occurred 1 day later. By Day 28, the bystander effect subsides and RBC recovery restarts at a rate of approximately 8100 RBCs/h/µL, which led to full recovery of the RBC numbers by Day 40. Within the period between Days 28 and 40, reticulocytes seem to rise again during Days 33–34. This peak could simply have been an error or noise in the determination, or due to a combination of biological events. In the latter case, since this peak is not accompanied by an increased RBC production, one of two explanations seems most likely: either the reticulocyte maturation time increased and decreased briefly, or a simultaneous increase in RBC production and loss due to bystander effect occurred. Neither one seems likely at this point of the infection, as the macaque had been curatively treated by Day 26 and no parasitaemia was recorded since then. Furthermore, given that the peak is rather short, it is not very likely due to a release of younger reticulocytes.

To explore the situation further, a slightly altered model was created, where it was assumed that a peak of production of RBCs and loss due to bystander effect occurred simultaneously and with the same magnitude (Fig. 5b, d); output from this model is shown in dotted lines in Fig. 5a, b, d. While the results are satisfactory, the explanation of two simultaneous processes with the same magnitude seems unlikely, and this model will, therefore, not be explored further.

During the period between Days 40 and 48, the erythropoietic output returns to its normal value. However, after Day 49 the model predicts a small drop in the erythropoietic output to 1740 RBCs/h/µL. This decrease reflects the decrease in RBC deaths by senescence, relative to the start of the infection. After Day 20, senescent cell death (Fig. 5e) decreases due to a changed age profile of RBCs (Additional file 2). Namely, during the infection large numbers of RBCs of all age classes are killed and subsequently replaced by newly produced RBCs. This replacement means that the older age classes are, after Day 50, notably less populated than under normal conditions (Additional file 2). Thus, until the age class profile is restored to its normal state, fluctuations in the senescent death rate are expected to occur for some time after the infection. Nevertheless, this drop in erythropoietic output, which is predicted after Day 49, also causes a small drop in reticulocytes and, indeed, the experimental data show a similar drop in reticulocytes numbers (Fig. 5a).

### Modelling RMe14, RSb14, and RIc14

The response profiles of the remaining macaques to the infections are documented as supplemental material (Additional file 3).

### Pre-patent increase in reticulocyte numbers

**Fig. 6:**
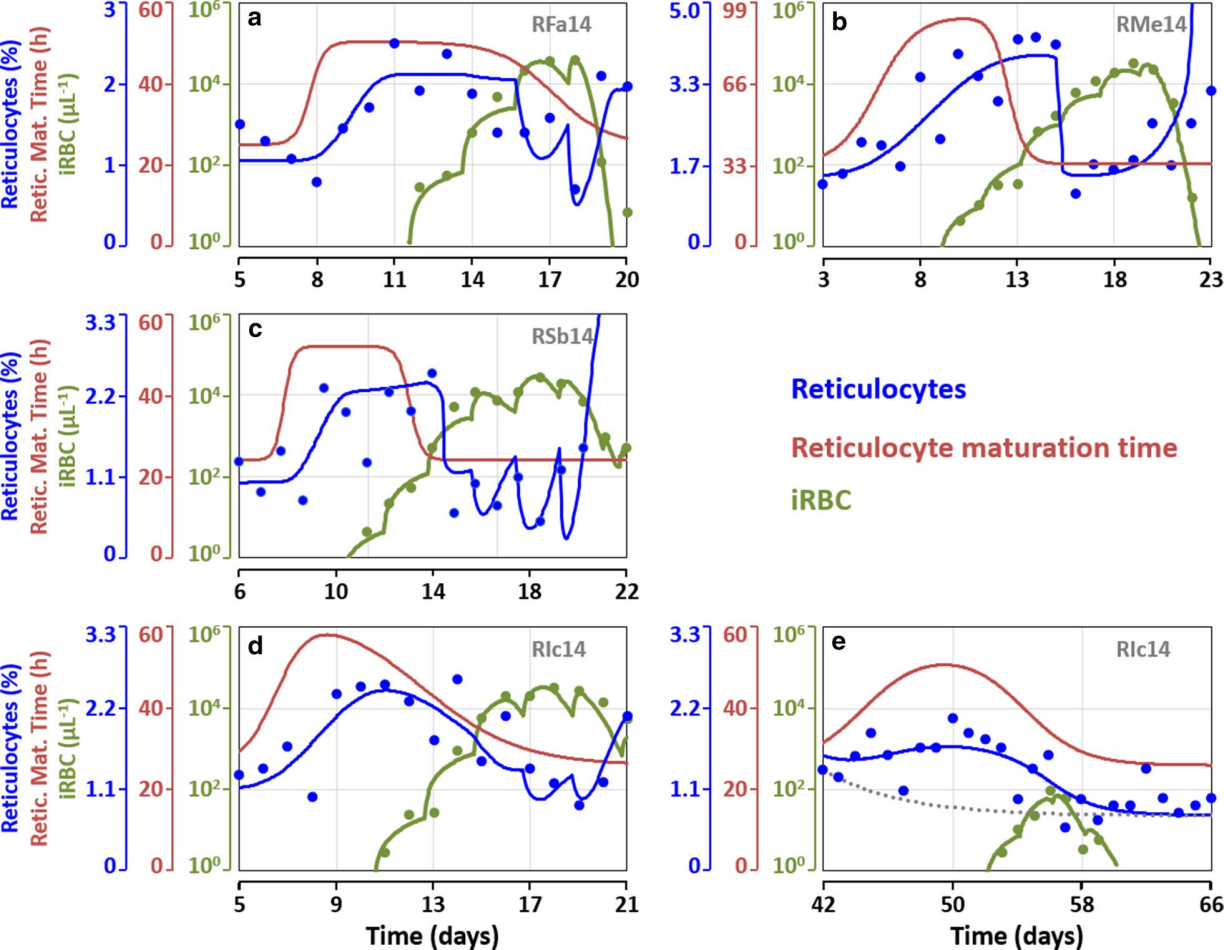
Comparison of the reticulocyte response during the first few days of infection. Experimental data for reticulocytes and infected RBCs are compared across the four macaques. **a** RFa14; **b** RMe14; **c** RSb14; **d** primary peak of infection for RIc14; **e** relapse infection for RIc14. In **e**, the dotted gray line depicts the simulation results obtained under the assumption that the maturation time of reticulocytes remains unchanged at 26 h and highlights the need to assume that an increase in the maturation is required to fit the experimental data. The best fitted models for each macaque are shown here, where each assumes a different reticulocyte-RBC preference ratio (RFa14 15:1; RMe14 1:1; RSb14 100:1; RIc14 10:1). In all cases, the predicted reticulocyte maturation time is at or close to its maximum when parasitaemia becomes patent, and so are the levels of reticulocytes needed to feed the ever growing number of parasites. The unknown mechanism or signal causing this increase in the reticulocyte maturation time seems to be occurring at the point where the blood infection starts between Days 5 and 7

All macaques modelled here showed evidence of an increase in reticulocyte numbers early in the infection (Fig. 5, Additional file 3: Figs. S2.1, S2.2, S2.3). Replotting these observations for all cases shows more clearly that the reticulocyte numbers increased before the infection patency (Fig. 6). The increases averaged at 1.7 ± 0.6%, with RFa14 increasing from 1.05 to 2.12% (Fig. 6a), RMe14 from 1.43 to 3.87% (Fig. 6b), RSB14 from 1.0 to 3.1% (Fig. 6c) and RIc14 from 1.1 to 2.44% (Fig. 6d). An analogous increase in reticulocytes was also detected in RIc14 just before the relapse infection peak (Fig. 6e). At the beginning of this time-period, between Days 42 and 66, RIc14 had recovered from the anaemia caused by the primary infection, and the erythropoietic production had been reduced (Additional file 3: Fig. S2.3B) to compensate for the lower rate of senescent RBC removal. The predicted levels of reticulocytes in the absence of an increase in reticulocyte maturation time in circulation are shown in panel E in gray (Fig. 6). This gray line clearly misses all data points, demonstrating that it is, indeed, necessary to assume an increase in the maturation time of reticulocytes in circulation between Days 42 and 58. Accounting for this increased maturation time in circulation leads to an increased percentage of peripheral blood reticulocytes from 0.87% (grey line) to 1.70% (blue line) (Fig. 6e).

If these increases in reticulocyte numbers in peripheral blood are caused by an increase in their maturation time in circulation, then the cause of this increase must precede it. In this model, the age of the reticulocytes released from the bone marrow is given by *ARR*_*t*_ (Eq. 1). It is the decrease of this quantity, representing the decrease in the age of newly released reticulocytes, which ultimately leads to the increase in their maturation time and the accumulation of reticulocytes in circulation. On average, the age of reticulocytes entering circulation starts to decrease 4 days before the detection of the infection by microscopy (7 days post-infection), at which time the parasitic infection is either still restricted to the liver or just beginning to be present in the blood.

### RBC fate during *P. cynomolgi* infection

**Fig. 7:**
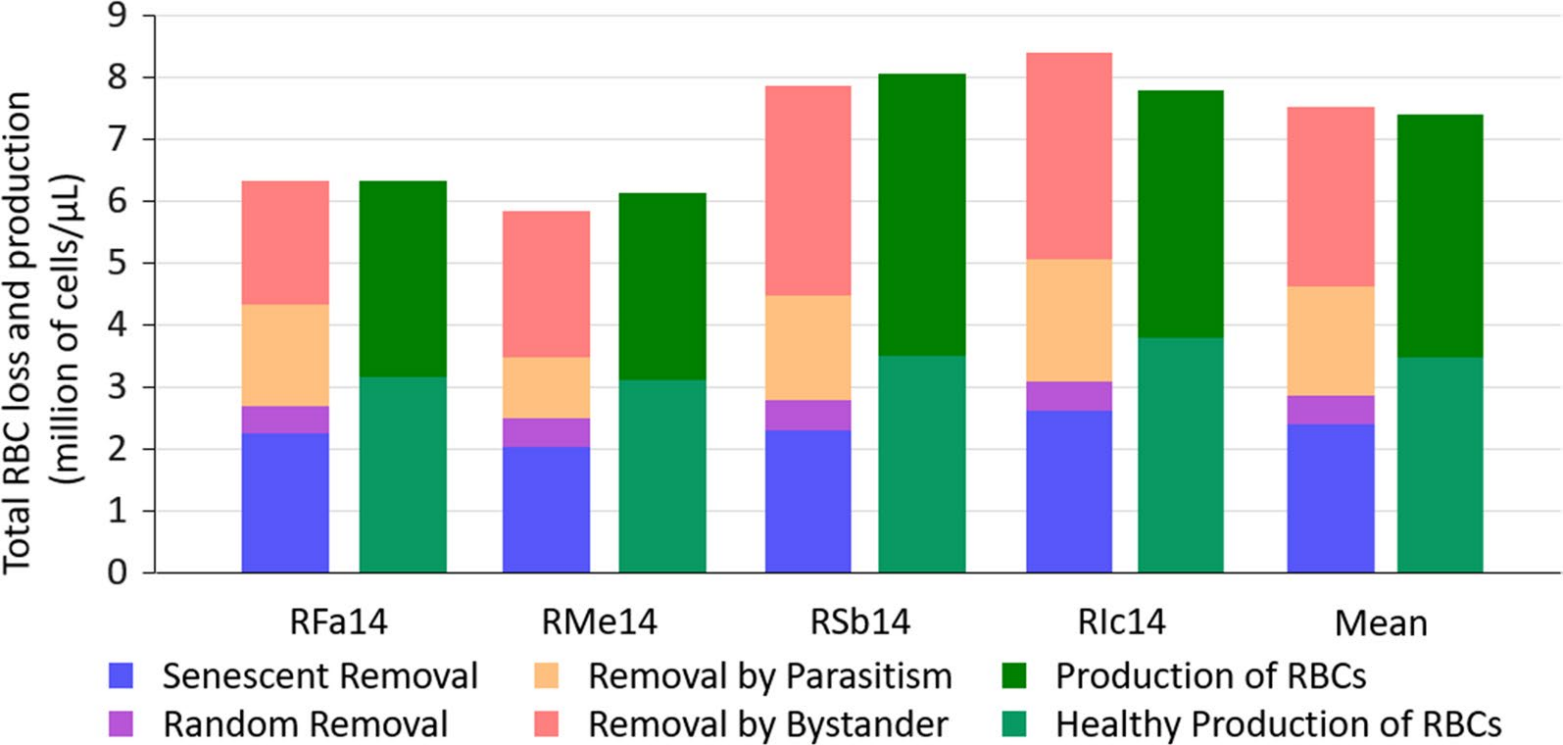
Comparison of the levels of RBC production and removal among ‘E04’ macaques. Total extent of RBC production and removal per macaque and for the average of RFa14, Rsb14 and RIc14 during the first 50 days of infection is shown. RMe14 was excluded from the average as this macaque received a blood transfusion that invalidates the true quantification of RBC production and removal. The extent of RBC production each macaque would have had if it had remained in its healthy state, shown during the first few days is highlighted with a lighter shade of green, out of the total measured RBC production. RBC removal is shown per process. The normal physiological processes are senescence and random loss. Infection-induced pathological processes are parasitism (invasion by the parasite) and bystander effect (loss of uninfected RBCs). Throughout the first 50 days of the infections, which include the primary parasitaemia peak but exclude relapses, 38 ±4% of RBCs were lost by the physiological processes (senescence and random loss), 38 ±6% due to bystander effect, and only 23 ±2% due to invasion by the parasite

The infection response profiles inferred for each macaque (Fig. 5 and Additional file 3: Figs. S2.1, S2.2, S2.3) were analysed for RBC production and removal, and the results are summarized in Fig. 7. Since these profiles are time-dependent and different for each macaque, the number of RBCs removed by each process was summed for the first 50 days of each infection, which in all cases includes the onset of the infection, anaemia, and recovery, but excludes relapses. RMe14 was excluded from the average as he received a blood transfusion, which clearly changed his reticulocyte and RBC profiles (Additional file 3: Fig. S2.1), and consequently would have invalidated the quantification of the haematological processes. If these macaques had not been infected but remained healthy with the same trajectory shown at their baselines, they would have produced, during these 50 days, an average of 3.5 ± 0.3 million RBCs/µL, of which 10% would have died by random processes and the remaining 90% by senescence. Yet, in every case, these infections resulted in the removal of twice as many RBCs, with an average of 7.6 ± 0.9 million RBCs/µL. Fewer RBCs were lost by the normal physiological processes (random and senescent), since many more were prematurely killed by the infection dependent processes. Interestingly, similar to what has been seen before in *Plasmodium coatneyi* infections [38], of the total number of RBCs removed due to the infection, only 38 ± 6% were actually killed by parasite invasion. The remaining 62 ± 8% were removed by the bystander effect. In contrast, *P. coatneyi* had 5% removed by parasite invasion and 95% by bystander effect [38].

### Dependence of the erythropoietic production on the severity of the anaemia

**Fig. 8:**
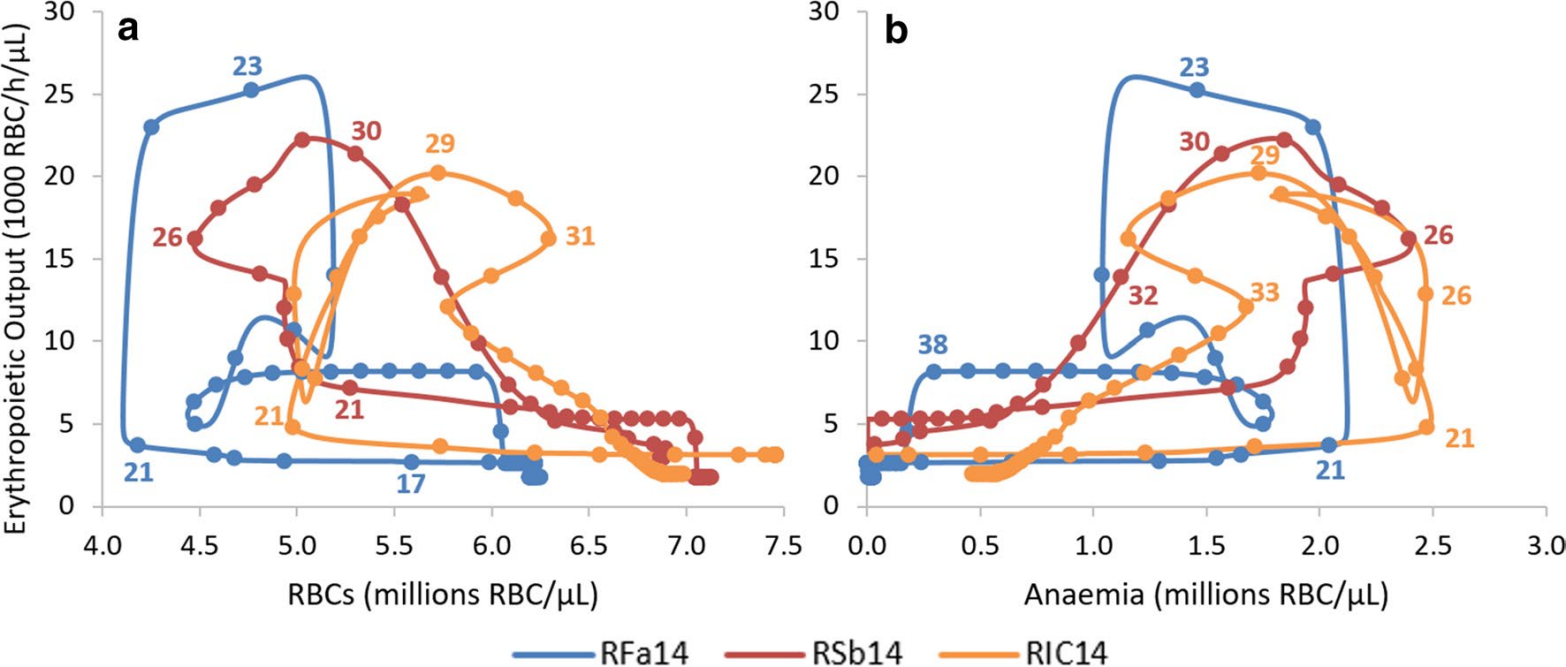
Dependence of the erythropoietic output on the levels of RBCs or on the severity of the anaemia. The erythropoietic output determined for each macaque is plotted against the RBC levels (**a**) and against the severity of the anaemia (**b**) for the same time points. Anaemia is calculated as the difference between the healthy RBC levels and the current level of RBCs of each macaque. Solid circles identify each experimental day, and some days are labelled with the corresponding time-stamp (in days post-infection) to allow visualization of the time dependence and direction. It is evident that there is no clear, direct correlation between the erythropoietic output and the number of RBCs

The level of erythropoietic output in the model, i.e., the rate of RBC production, was inferred from each macaque’s profile data (Fig. 5, Additional file 3: Figs. S2.1, S2.2, S2.3). Since this property is not being assumed to be explicitly dependent on the severity of the anaemia, nor on the number of RBCs, it becomes possible to test these dependencies and the underlying assumptions. Figure 8 shows the erythropoietic output of each macaque, plotted against the RBC levels (Fig. 8a) and against anaemia (Fig. 8b). Here, anaemia represents the number of missing RBCs relative to the healthy state of each macaque. Therefore, periods where the macaques had very mild or no anaemia (Fig. 8b) correspond to periods with high levels (6–7.5 million) of RBCs (Fig. 8a).

A usual assumption in the field is that the erythropoietic output decreases as the RBC levels approach a healthy value, which is to say that the erythropoietic output is negatively correlated with RBC levels and a negative trend would be visible in panel A. Similarly, a positive correlation would be found in panel B if the RBC production increased with the severity of the anaemia. Yet, such correlations are not present. One possible explanation for this lack of correlation could be that there is a time lag between the onset of the anaemia and the increase in the RBC production, as it takes time for the kidneys to detect the low levels of oxygen in the blood, produce erythropoietin, allow this hormone to travel to the bone marrow, exert its effect on RBC progenitors, and actually to increase the release of reticulocytes. Different time lags were tested and none resulted in a correlation being detected in either case, which suggests a couple of possible explanations. The response could be highly non-linear, and/or the presence of a malarial infection may interfere with the bone marrow response to such a degree that the correlation disappears. Unsurprisingly, bone marrow dysfunction has been previously reported for this cohort of animals, when infected with *P. cynomolgi* [36].

## Discussion

Nonhuman primate model systems hold the greatest potential for understanding malarial host-parasite dynamics and pathogenesis in vivo [14, 45–47]. A new computational model is presented here for the analysis of erythrocyte dynamics during infections with *P. cynomolgi*, a relapsing simian malaria parasite that is a zoonosis and can serve as an experimental model for *P. vivax*. This computational model was parameterized to reproduce experimental data obtained from *P. cynomolgi* infection of Rhesus macaques [17] and allowed the characterization of the lifespan of reticulocytes in healthy malaria-naïve animals, the interactions between parasite and host, and the host responses during a blood-stage infection.

Process quantification in this model was done by inference of a time-dependent function from the experimental data, rather than assuming that these processes follow a mass-action model in an *ad*-*hoc* fashion, as it is commonly done in the field [30–33]. By avoiding the assumption of such relationships, it becomes possible to test assumptions and system properties in an unbiased manner. One such property examined was the erythropoietic output, which corresponds to the rate of RBC production and the rate of reticulocyte release from the bone marrow. This property is usually assumed to depend linearly on present or recent anaemia or to be inversely proportional to the present or recent RBC levels [48, 49]. Yet, the results show no correlation between either, which suggests that the true function is more complex and likely includes delays.

In humans, the maturation time of reticulocytes in circulation is about 24 h [50, 51], but this period may be increased to as many as 3 days under erythropoietic stress [52]. Here, the computational model shows that the maturation time of circulating reticulocytes in healthy *Macaca mulatta* is about 24 ± 5 h (n = 15). This value is corroborated by an in vitro analysis of the surviving reticulocytes in fresh RBC cultures from healthy macaques. In these cultures, reticulocytes remained detectable for 25 h, as identified by their RNA content. Overall, these results point to a similar reticulocyte kinetics between *Macaca mulatta* and humans. From the same analysis, the normal healthy RBC production rate was determined to be 2727 ± 209 cells/h/µL (n = 15) for *Macaca mulatta*.

All macaques exhibited a period of elevated reticulocyte levels 11–13 days after the inoculation with sporozoites, which preceded the detection of patent parasitaemia in the blood and occurred concurrently without any change in RBC numbers. Theoretically, an increase in reticulocyte numbers can only be due to one of two processes, or both: (1) increased RBC production and release from the bone marrow; or (2) a shift towards the release of younger reticulocytes, as it has been observed during erythropoietic stress [52]. The first would lead to increased reticulocyte numbers in circulation, along with an increase in the overall RBC numbers. In the latter, the same total number of RBCs is still being produced, so no increase in RBC numbers would be observed. However, if reticulocytes are released at a younger stage from the bone marrow, these would take a longer time to mature in circulation, thus leading to an increased accumulation of reticulocytes in circulation. Given that an increase in the total number of RBCs was not observed, an increase in the RBC production does not explain the current data. Therefore, the more likely explanation is a shift toward the release of less mature reticulocytes. Under this assumption, this shift would have to occur around days 6–8 to be consistent with the observed changes, which puts this shift at around the time the parasites are coming out of the liver and starting the infection’s blood stage. A plausible explanation may be that during the beginning of the blood-stage infection the parasite releases a factor that ultimately results in the release of younger reticulocytes from the bone marrow. This mechanism would ensure an increase of circulating reticulocytes, which are arguably the parasite’s preferred host cells, although the preference is not exclusive [8, 24, 25]. The increased number of reticulocytes seems to subside before a subsequent reticulocyte peak occurs due to the host’s response to the anaemia. During the high parasitaemia period, the reticulocyte numbers exhibit oscillations. These seem to be due to the cycles of RBC infection. In this period of the infection, it is difficult for the model to distinguish precisely between the high consumption of reticulocytes and the decrease in maturation time of these cells. However, the decrease in maturation time has to happen during this time period, because the model only fits the reticulocyte peak in response to the anaemia if the reticulocyte maturation time has returned to normal. These observations are interesting as they suggest that *P. cynomolgi* parasites may be capable of causing a shift in the age at which reticulocytes are released from the bone marrow. This shift may be advantageous for the parasite as it happens in anticipation of the parasite’s high demand for reticulocytes and, secondarily, red blood cells, and occurs a week after the release from the liver, when the parasite numbers reach their maximum.

It has long been recognized that certain *Plasmodium* species show preferences for invading mature RBCs or reticulocytes [25, 53]. Species like *Plasmodium ovale* and *P. vivax* have almost strict reticulocyte tropism, whereas *P. coatneyi*, *Plasmodium knowlesi* and *Plasmodium malariae* have mature RBC tropism [24]. By contrast, *P. falciparum* invades both mature and immature RBCs. *P. cynomolgi* resembles *P. vivax*, which has an almost strict reticulocyte tropism [24], whereas the tropism appears to be conditional in the case of *P. cynomolgi* [25, 54], thus exhibiting a preference for reticulocytes while maintaining the ability to infect both RBC maturation stages. Using the computational model developed here, the reticulocyte preference calculated for each macaque is about 15, 1, 100 and 10 (RFa14, RMe14, RSb14 and RIc14, respectively), which gives an average of 32 ± 46 (n = 4) or a median of 13 for this cohort. These results are rough estimates, as they depend highly on the level of reticulocytes during peak parasitaemia, a short time span with just about six time points for each macaque. Additionally, the reticulocyte preference parameter also suffers from structural correlation with the reticulocyte maturation time. The model results show that the reticulocyte maturation and release timing returns to normalcy by the time high parasitaemias are observed, which allows averting the issue of structural non-identifiability. The value determined here for the reticulocyte preference is lower than what was measured for *Plasmodium berghei*, 153 [55], and closer to what was recently obtained for *P. berghei* ANKA strain, 74 [56]. Additionally, a recent in vitro study showed that *P. cynomolgi* B strain has strict tropism towards human reticulocytes, but this was not evident when testing *Macaca mulatta* RBCs [8]. Unfortunately, this study did not address possible host cell preferences using co-cultures of reticulocytes and RBCs, thus preventing the quantification of *P. cynomolgi* preference for *Macaca mulatta* reticulocytes. Overall it appears that the reticulocyte preference is an important parameter for the infection dynamics, as it has been shown that the preference may be correlated with parasitaemia levels and ultimately with disease severity [33, 48, 49].

Analysis of the parasite growth rates within each macaque revealed that the parasite population grew at unexpectedly fast rates, mostly with values in the range of 30–54 merozoites per infected RBC. In the case of the *P. cynomolgi* infected macaque RFv13, the computed value actually reached 110 merozoites per infected RBC, which is likewise unrealistic. This monkey had a particularly high peak parasitaemia (19.5%), suffered severe manifestations of the disease and ultimately needed to be euthanized [7, 17]. A likely explanation of the apparently high growth rates is that not all parasite forms circulate freely but may rather become concealed in venules or tissues [44], such that parasitaemia readings from peripheral blood smears may not reflect the total parasite load in the blood. If indeed a substantial number of infected RBCs go into concealment for some of their 48-h life cycle, then the parasite population based on blood smear readings may at times appear to grow at a faster rate than what is biologically possible. This hypothesis has been analysed for *P. cynomolgi*, where the analysis of in vivo data suggested the existence of a population of non-circulating concealed parasites [44]. The model here did not consider concealment and accounted only for parasites observed on blood smears. However, as compensation, the parasitaemias were allowed to grow at the observed, seemingly inflated, growth rates, even though these are higher than what would be biologically possible. In this way, the growth of the parasitaemias does take into consideration any non-visible, and thus concealed, parasites, without requiring any assumption regarding possible concealment probabilities, kinetics, or sites.

Analysis of the infection profiles of RFa14 and RIc14 points to a temporal segregation of RBC production and removal (Fig. 5 and Additional file 3: Fig. S2.3), and similar results were found for *P. coatneyi* [38]. Interestingly, RSb14 (Additional file 3: Fig. S2.2) does not fit this pattern, as it shows an increased level of RBC production that lasts 15 days with its maximum at Day 30, during which time losses due to bystander effect and parasite invasion are recorded. Given the limited sample size, all cases are being reported here. In addition to the deconvolution of loss and production of RBCs in RFa14 and RIc14, RBC losses by parasite invasion and bystander effect tend not to occur simultaneously. This observation gives confidence that loss due to bystander effect is real and not due to a miss-calculation of parasite invasion. Additionally, removal by bystander effect is also detected in periods where parasitaemia is low, which further strengthens this point. Yet, the fact that these two losses tend to be segregated from RBC production does suggest that something involved with the RBC losses prevents up-regulation of the erythropoietic system even during periods of anaemia.

For example, the profile of RIc14 contains a period (Days 23–28) where the main parasitaemia peak had subsided, and the erythropoietic production is high. Suddenly, parasitaemia increases again, and the model measures an increase in RBC death, due to both invasion and bystander effect, which is accompanied by a decrease in RBC production. The decrease in production is inferred by the model as a result of the decrease in reticulocyte counts. What the mediator of this effect could be is not known, but the unknown factor could be mediated by the immune system, as suggested by the analysis of the bone marrow transcriptome of this macaque cohort [36]. Additionally, it is possible that some aspect of the immune response may be the culprit for the bystander effect, which would simultaneously explain uninfected and infected RBC losses and the failure to up-regulate RBC production.

However, the analysis never shows a reduction of the erythropoietic flux during the infection and recovery periods. The healthy baseline RBC production is determined from the RBC status of each macaque during the first 5–7 days of the experiment, and across all infections, the RBC production is never inferred to dip below this healthy level. Thus, anaemia cannot be due to decreased RBC production, but is more likely due to increased RBC destruction, either by parasite invasion and a bystander effect, and to suppression of the erythropoietin-dependent up-regulation of erythropoiesis in response to anaemia, despite elevated levels of erythropoietin in this macaque cohort between Days 20 and 30 [36]. By contrast, decreased RBC production is observed only after full recovery of the macaques, but that is due to a shift in the age distribution of RBCs which becomes skewed toward a younger than normal population (see Additional file 1). This younger population of RBCs is, therefore, subject to fewer losses due to old age, and the erythropoietic system of these macaques transiently adjusts the production to meet these reduced losses at normal haematocrit levels.

The bystander effect was estimated from all RBCs produced and lost throughout the first 50 days of the infection. This period of 50 days post-inoculation includes the main parasitaemia peak, anaemia, treatment if needed, and recovery, but excludes relapses. As a consequence of the infections, RBC production and removal doubled to 217% (from 3.5 ± 0.3 to 7.6 ± 0.9 million RBCs*/*µL). During this same period, RBC removal occurred due to normal physiological processes of senescence and random loss (38 ± 4%), invasion by the parasite (23 ± 2%), and the bystander effect (38 ± 6%). These results suggest that the bystander effect accounts for 62 ± 8% of all infection-induced RBC losses.

Bystander removal of RBCs during malaria has been documented in humans [41, 57, 58], yet accurate measurements are difficult to obtain [41]. Using a mathematical model similar to the one used here, the bystander removal of RBCs was inferred for *Macaca mulatta* during *P. coatneyi* infections as 95% [38], which is similar to values estimated for humans with falciparum malaria (90–92%) [41, 58]. Bystander loss of RBCs has also been documented in vivax malaria [58] and may be due to changes in membrane rigidity, although other mechanisms are under investigation [59, 60]. The present data do not allow inferences regarding the possible underlying causes of the bystander effect, but they do show that proportionally fewer RBCs are removed by the bystander effect in *P. cynomolgi* (62%) infections than in *P. coatneyi* (95%) [44]. Whether this difference is indicative of the difference between the human counterparts of these infections (vivax and falciparum, respectively) is yet to be determined.

## Conclusions

Nonhuman primate models of malaria are as close to human malaria as possible, and much can be inferred regarding the disease progression in humans from NHP models. Here, the dynamics of reticulocytes and RBCs was investigated with a new mathematical model. This model uses a discrete recursive framework with age-structure, which allows the estimation of the healthy maturation time of reticulocytes in circulation and of normal RBC production in Rhesus macaques. The maturation time was determined as 24 ± 5 h (*n *= 15), and RBC production as 2727 ± 209 cells/h/µL (*n *= 15). The reticulocyte maturation time in circulation was validated in ex vivo experiments and is similar to that in humans. Analysis of the responses of Rhesus macaques to *P. cynomolgi* infections revealed a period during the early blood-stage of the infection when the numbers of reticulocytes in the peripheral blood increase. The model analysis suggests that this short period comprising the early rise in blood-stage parasitaemia may be associated with reticulocyte tropism of this *Plasmodium* species, which would be consistent with previous indications of host cell preferences by *P. cynomolgi* [25]. The results also suggest a temporal segregation between RBC producing and RBC removing processes, which points towards the existence of some unidentified factor, which may prevent up-regulation of erythropoiesis during anaemia and while RBCs are being destroyed either by invasion or due to the bystander effect. The main infection peak and subsequent recovery resulted in an overall doubling (214%) of the number of RBCs produced relative to what would be expected in the absence of infection. Of the total number of RBCs lost due to these infections, 62% were lost by processes other than parasitic invasion, here designated as the bystander effect. This value is significantly lower than the value of 95% that was obtained previously for *P. coatneyi* infections in rhesus macaques [38].

## Additional files

**Additional file 1.** Age dependent invasion of RBCs.

**Additional file 2.** RBC age distribution at day 0 and day 50 for RFa14.

**Additional file 3.** Profiles for RMe14, RSb14, and RIc14.

## References

[1] WHO (2015). Control and elimination of *Plasmodium vivax* malaria—a technical brief.

[2] Borner J, Pick C, Thiede J, Kolawole OM, Kingsley MT, Schulze J (2016). Phylogeny of haemosporidian blood parasites revealed by a multi-gene approach. Mol Phylogenet Evol.

[3] Baird JK (2013). Evidence and implications of mortality associated with acute *Plasmodium vivax* malaria. Clin Microbiol Rev.

[4] Howes RE, Battle KE, Mendis KN, Smith DL, Cibulskis RE, Baird JK (2016). Global epidemiology of *Plasmodium vivax*. Am J Trop Med Hyg.

[5] Pacheco MA, Battistuzzi FU, Junge RE, Cornejo OE, Williams CV, Landau I (2011). Timing the origin of human malarias: the lemur puzzle. BMC Evol Biol.

[6] Pasini EM, Bohme U, Rutledge GG, Voorberg-Vander Wel A, Sanders M, Berriman M (2017). An improved *Plasmodium cynomolgi* genome assembly reveals an unexpected methyltransferase gene expansion. Wellcome Open Res..

[7] Joyner C, Consortium TM, Wood JS, Moreno A, Garcia A, Galinski MR (2017). Severe and complicated cynomolgi malaria in a Rhesus macaque resulted in similar histopathological changes as those seen in human malaria. Am J Trop Med Hyg..

[8] Kosaisavee V, Suwanarusk R, Chua ACY, Kyle DE, Malleret B, Zhang R (2017). Strict tropism for cd71(+)/cd234(+) human reticulocytes limits the zoonotic potential of *Plasmodium cynomolgi*. Blood.

[9] Imwong M, Madmanee W, Suwannasin K, Kunasol C, Peto TJ, Tripura R (2018). Asymptomatic natural human infections with the simian malaria parasites *Plasmodium cynomolgi* and *Plasmodium knowlesi*. J Infect Dis..

[10] Medica DL, Sinnis P (2005). Quantitative dynamics of *Plasmodium yoelii* sporozoite transmission by infected anopheline mosquitoes. Infect Immun.

[11] Ponnudurai T, Lensen AH, van Gemert GJ, Bolmer MG, Meuwissen JH (1991). Feeding behaviour and sporozoite ejection by infected *Anopheles stephensi*. Trans R Soc Trop Med Hyg.

[12] Prudencio M, Rodriguez A, Mota MM (2006). The silent path to thousands of merozoites: the *Plasmodium* liver stage. Nat Rev Microbiol.

[13] Vaughan AM, Aly AS, Kappe SH (2008). Malaria parasite pre-erythrocytic stage infection: gliding and hiding. Cell Host Microbe.

[14] Joyner C, Barnwell JW, Galinski MR (2015). No more monkeying around: primate malaria model systems are key to understanding *Plasmodium vivax* liver-stage biology, hypnozoites, and relapses. Front Microbiol..

[15] Krotoski WA, Collins WE, Bray RS, Garnham PC, Cogswell FB, Gwadz RW (1982). Demonstration of hypnozoites in sporozoite-transmitted *Plasmodium vivax* infection. Am J Trop Med Hyg.

[16] White NJ (2011). Determinants of relapse periodicity in *Plasmodium vivax* malaria. Malar J..

[17] Joyner C, Moreno A, Meyer EV, Cabrera-Mora M, Ma HC, Kissinger JC (2016). *Plasmodium cynomolgi* infections in Rhesus macaques display clinical and parasitological features pertinent to modelling *vivax* malaria pathology and relapse infections. Malar J..

[18] Coatney GR, Allergy NIO (1971). Diseases I: the primate malarias.

[19] Murphy GS, Oldfield EC (1996). Falciparum malaria. Infect Dis Clin North Am.

[20] Genton B, D’Acremont V (2001). Clinical features of malaria in returning travelers and migrants.

[21] Rahimi BA, Thakkinstian A, White NJ, Sirivichayakul C, Dondorp AM, Chokejindachai W (2014). Severe vivax malaria: a systematic review and meta-analysis of clinical studies since 1900. Malar J..

[22] WHO (2000). Severe falciparum malaria. Trans R Soc Trop Med Hyg.

[23] WHO (2014). Severe malaria. Trop Med Int Health.

[24] Russell BM, Cooke BM (2017). The rheopathobiology of *Plasmodium vivax* and other important primate malaria parasites. Trends Parasitol..

[25] Warren M, Skinner JC, Guinn E (1966). Biology of the simian malarias of southeast Asia. I. Host cell preferences of young trophozoites of four species of *Plasmodium*. J Parasitol..

[26] Akinyi S, Hanssen E, Meyer EVS, Jiang J, Korir CC, Singh B (2012). A 95 kDa protein of *Plasmodium vivax* and *P. cynomolgi* visualized by three-dimensional tomography in the caveola vesicle complexes (Schüffner’s dots) of infected erythrocytes is a member of the phist family. Mol Microbiol..

[27] Aikawa M, Miller LH, Rabbege J (1975). Caveola–vesicle complexes in the plasmalemma of erythrocytes infected by *Plasmodium vivax* and *P. cynomolgi.* Unique structures related to Schüffner’s dots. Am J Pathol..

[28] Mideo N, Day T, Read AF (2008). Modelling malaria pathogenesis. Cell Microbiol.

[29] Khoury DS, Aogo R, Randriafanomezantsoa-Radohery G, McCaw JM, Simpson JA, McCarthy JS (2018). Within-host modeling of blood-stage malaria. Immunol Rev.

[30] Anderson RM, May RM, Gupta S (1989). Non-linear phenomena in host—parasite interactions. Parasitology.

[31] Hetzel C, Anderson RM (1996). The within-host cellular dynamics of bloodstage malaria: theoretical and experimental studies. Parasitology.

[32] Johnson PLF, Kochin BF, Ahmed R, Antia R (2012). How do antigenically varying pathogens avoid cross-reactive responses to invariant antigens?. Proc R Soc Lond B Biol Sci.

[33] Mcqueen PG, Mckenzie FE (2006). Competition for red blood cells can enhance *Plasmodium vivax* parasitemia in mixed-species malaria infections. Am J Trop Med Hyg.

[34] Fonseca LL, Voit EO (2015). Comparison of mathematical frameworks for modeling erythropoiesis in the context of malaria infection. Math Biosci.

[35] Access data from mahpic—the malaria host-pathogen interaction center. http://plasmodb.org/plasmo/mahpic.jsp.

[36] Tang Y, Joyner CJ, Cabrera-Mora M, Saney CL, Lapp SA, Nural MV (2017). Integrative analysis associates monocytes with insufficient erythropoiesis during acute *Plasmodium cynomolgi* malaria in Rhesus macaques. Malar J..

[37] Schirm S, Engel C, Loeffler M, Scholz M (2013). A biomathematical model of human erythropoiesis under erythropoietin and chemotherapy administration. PLoS ONE.

[38] Fonseca LL, Alezi HS, Moreno A, Barnwell JW, Galinski MR, Voit EO (2016). Quantifying the removal of red blood cells in *Plasmodium coatneyi* infection. Malar J..

[39] Moreno A, Cabrera-Mora M, Garcia A, Orkin J, Strobert E, Barnwell JW (2013). *Plasmodium coatneyi* in Rhesus macaques replicates the multisystemic dysfunction of severe malaria in humans. Infect Immun.

[40] Dasari P, Fries A, Heber SD, Salama A, Blau I-W, Lingelbach K (2014). Malarial anemia: digestive vacuole of *Plasmodium falciparum* mediates complement deposition on bystander cells to provoke hemophagocytosis. Med Microbiol Immunol.

[41] Jakeman GN, Saul A, Hogarth WL, Collins WE (1999). Anaemia of acute malaria infections in non-immune patients primarily results from destruction of uninfected erythrocytes. Parasitology.

[42] Lichtman M, Beutler E, Kipps T, Seligsohn U, Kaushansky K, Prchal J (2010). Williams hematology.

[43] Lee KJ, Yin W, Arafat D, Tang Y, Uppal K, Tran V (2014). Comparative transcriptomics and metabolomics in a Rhesus macaque drug administration study. Front Cell Dev Biol..

[44] Fonseca LL, Joyner CJ, Consortium M, Galinski MR, Voit EO (2017). A model of *Plasmodium vivax* concealment based on *Plasmodium cynomolgi* infections in *Macaca mulatta*. Malar J..

[45] Langhorne J, Buffet P, Galinski M, Good M, Harty J, Leroy D (2011). The relevance of non-human primate and rodent malaria models for humans. Malar J..

[46] Ng S, March S, Galstian A, Gural N, Stevens KR, Mota MM (2017). Towards a humanized mouse model of liver stage malaria using ectopic artificial livers. Sci Rep..

[47] Vallender EJ, Miller GM (2013). Nonhuman primate models in the genomic era: a paradigm shift. ILAR J.

[48] Antia R, Yates A, Roode JCD (2008). The dynamics of acute malaria infections. I. Effect of the parasite’s red blood cell preference. Proc R Soc Lond B Biol Sci..

[49] Mcqueen PG, Mckenzie FE (2004). Age-structured red blood cell susceptibility and the dynamics of malaria infections. Proc Natl Acad Sci USA.

[50] Kaushansky K, Lichtman MA, Beutler E, Kipps TJ, Seligsohn U, Prchal JT (2011). Williams hematology.

[51] Skadberg O, Brun A, Sandberg S (2003). Human reticulocytes isolated from peripheral blood: maturation time and hemoglobin synthesis. Lab Hematol..

[52] Brugnara C (1998). Use of reticulocyte cellular indices in the diagnosis and treatment of hematological disorders. Int J Clin Lab Res.

[53] Craik R (1920). The erythrocytes in malaria. Lancet.

[54] Sutton PL, Luo Z, Divis PCS, Friedrich VK, Conway DJ, Singh B (2016). Characterizing the genetic diversity of the monkey malaria parasite *Plasmodium cynomolgi*. Infect Genet Evol..

[55] Cromer D, Evans KJ, Schofield L, Davenport MP (2006). Preferential invasion of reticulocytes during late-stage *Plasmodium berghei* infection accounts for reduced circulating reticulocyte levels. Int J Parasitol.

[56] Thakre N, Fernandes P, Mueller A-K, Graw F (2017). Characterizing malaria blood-stage infection patterns of two *Plasmodium* parasite strains. Front Microbiol..

[57] Collins WE, Jeffery GM, Roberts JM (2003). A retrospective examination of anemia during infection of humans with *Plasmodium vivax*. Am J Trop Med Hyg.

[58] Price RN, Simpson JA, Nosten F, Luxemburger C, Hkirjaroen L, ter Kuile F (2001). Factors contributing to anemia after uncomplicated falciparum malaria. Am J Trop Med Hyg.

[59] Handayani S, Chiu DT, Tjitra E, Kuo JS, Lampah D, Kenangalem E (2009). High deformability of *Plasmodium vivax*-infected red blood cells under microfluidic conditions. J Infect Dis.

[60] Paul A, Padmapriya P, Natarajan V (2017). Diagnosis of malarial infection using change in properties of optically trapped red blood cells. Biomed J..

