# Supplementary material for: Analysis of erythrocyte dynamics in Rhesus macaque monkeys during infection with Plasmodium cynomolgi
Source: Malar J. 2018 Nov 6;17:410. doi: 10.1186/s12936-018-2560-6 (PMC6219197; doi:10.1186/s12936-018-2560-6)
Supplement: Supplementary file 1 — Additional file 1. Age dependent invasion of RBCs. [file 12936_2018_2560_MOESM1_ESM.docx]

# Additional file 1

**Age dependent invasion of RBCs**

Invasion of RBCs follows an age-preference function (*RBCAPf_i_*, Eq. (21) of the main text). After invasion of all reticulocytes, *M_t_* − *NiRET* merozoites are still available to infect RBCs. The number of RBCs of each age class that are to be removed is given by:

|  | ${PiRBC}_{i}=\frac{{RBCAPf}_{i}\cdot{RBC}_{i,t}}{\sum_{j=i}^{3840} {RBCAPf}_{j}\cdot{RBC}_{j,t}}, i\in\left\{ 1, 2, \ldots, 3840 \right\}.$ | (1.1) |
| --- | --- | --- |

An iterative process is used to ensure that no more than the available number of RBCs in each age class is removed. First, the remaining number of merozoites is defined as:

|  | $MerLeft=M_{t}-iRET.$ | (1.2) |
| --- | --- | --- |

Let *f* = 1*...*3840 with a step of 1, and

|  | ${NiRBC}_{f}=\left\{ \begin{matrix} MerLeft\cdot{PiRBC}_{f}, & if MerLeft\cdot{PiRBC}_{f}<{RBC}_{f,t} \\ {RBC}_{f,t}, & if MerLeft\cdot{PiRBC}_{f}\geq{RBC}_{f,t} \end{matrix} \right..$ | (1.3) |
| --- | --- | --- |

The number of available merozoites is updated to:

|  | $MerLeft=M_{t}-NiRET-\sum_{i=1}^{f} {NiRBC}_{i}.$ | (1.4) |
| --- | --- | --- |

Eqs. (1.3) and (1.4) are iterated, until *f* reaches 3840. At that point, *NiRBC_i_* contains the number of RBCs to be removed from each age class. Therefore, the fraction of RBCs that are to be invaded is:

|  | ${RBCInv}_{i,t}=\frac{{NiRBC}_{i}}{{RBC}_{i,t}}, i\in\left\{ 1, 2, \ldots, 3840 \right\}.$ | (1.5) |
| --- | --- | --- |
