# Supplementary material for: Analysis of erythrocyte dynamics in Rhesus macaque monkeys during infection with Plasmodium cynomolgi
Source: Malar J. 2018 Nov 6;17:410. doi: 10.1186/s12936-018-2560-6 (PMC6219197; doi:10.1186/s12936-018-2560-6)
Supplement: Supplementary file 2 — Additional file 2. RBC age distribution at day 0 and day 50 for RFa14. [file 12936_2018_2560_MOESM2_ESM.docx]

Figure S1A. Age-distribution of RBCs at Days 0 and 50 for RFa14, inferred from experimental data. The hazard function is supplied for reference, as this function determines the percentage of RBCs removed by senescence per age bracket. Inspection of the Day-50 RBC distribution, in comparison to the healthy distribution at Day 0, shows that very few cells are left between the ages of 30 and 60 and that older cell age brackets (ages 60 to 100 days) are depleted as well. These cells were lost during the infection due to either invasion by the parasite or the bystander effect (Fig S2.1B). The peaks between the cell ages 10 and 30 were produced by the up-regulation of the erythropoietic output during the recovery period (Fig 5B). Interestingly, the areas under these curves (Day 0 and Day 50) are almost the same, approximately 6.1 million RBCs/µL of blood. Due to this shifted balance between young and old RBCs, there is less loss of RBCs due to old age at Day 50, simply because there are fewer old RBCs. In response to this state with less loss due to old age and normal levels of RBCs, the erythropoietic system adjusts to the decreasing RBC production, which leads to a slightly decreased level of reticulocytes seen in Fig 5A.

Figure S1B. Age distributions of RBCs at Days 0, 20, 40 and 60 for RFa14. The plot highlights what happens to the RBC age distribution as the macaque lives through the infection, during Days 0 to 20; recovery, Days 20 to 40; and post recovery, Days 40 to 60. During the infection period, large numbers of RBCs are destroyed and the comparison between the age distributions at Days 0 and 20 shows a large loss of young RBC cells (0-40 days old). At Day 40, these depleted brackets are now 20 days older (20-40 days old), but due to the recovery of the RBC numbers, the age brackets between 0 and 20 days have now more RBCs than normal. At day 60, the age brackets with high numbers of cells are 20 days older than at Day 40 and occupy the age brackets between 20 and 40 days of age. The peaks are slightly lower now, as some cells have been removed by random death. One can also see the result of the lowering of erythropoietic output, which occurs at Day 50 and causes the age brackets between 0 and 10 to be less populated than the age brackets between 10 and 20.
