# Supplementary material for: Analysis of erythrocyte dynamics in Rhesus macaque monkeys during infection with Plasmodium cynomolgi
Source: Malar J. 2018 Nov 6;17:410. doi: 10.1186/s12936-018-2560-6 (PMC6219197; doi:10.1186/s12936-018-2560-6)
Supplement: Supplementary file 3 — Additional file 3. Profiles for RMe14, RSb14, and RIc14. [file 12936_2018_2560_MOESM3_ESM.docx]

# Additional file 3:

**Characterization of the responses to the *Plasmodium* infections in RMe14, RSb14, and RIc14**

**Modelling RMe14**

Similar to RFa14, RMe14 presented no change from the initial phenotype for the first four days. This time period was used to infer a maturation time of reticulocytes in circulation of 34 hour and an erythropoietic output of 2,590 RBCs/h/µL (Fig. S2.1B). Around Day 5, the percentage of reticulocytes started to increase from 1.5% to 4.25% by Day 13. This rise was assumed to be due to a shift towards the release of less mature reticulocytes from the bone marrow. At its maximum, by Day 10, the inferred maturation time of reticulocytes rose to 93 hours (Fig. S2.1B). Although the reticulocyte maturation time increased to a higher value in this macaque, relative to RFa14, no increase in RBC numbers (Fig. S2.1C) was detected that could offer any other explanation as to the cause of the reticulocyte increase, just as in RFa14.


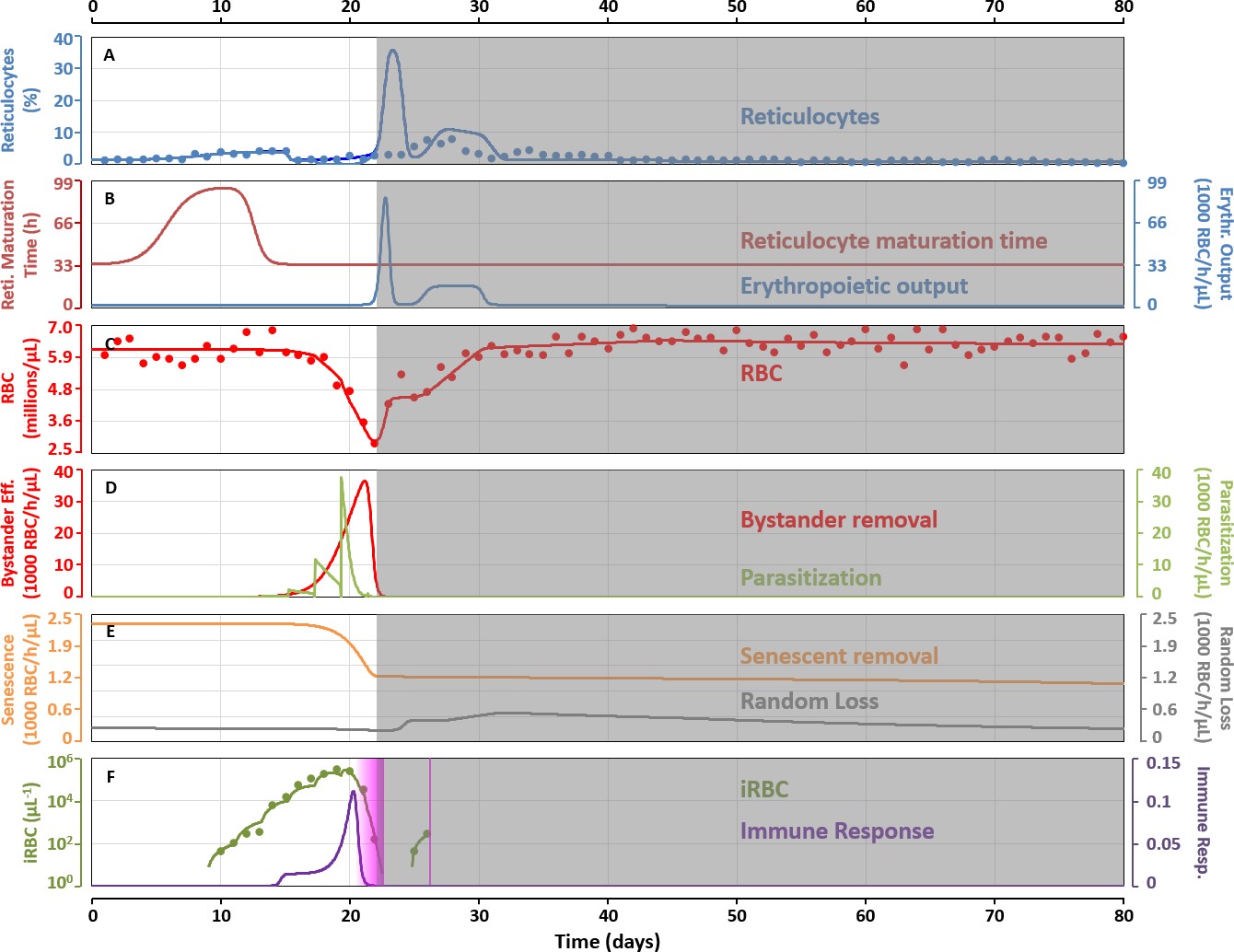


**Figure S2.1 Characterization of RMe14 infection.** Panels A, C and F show the experimentally obtained data for reticulocytes, RBCs, and infected RBCs, with the corresponding model fits. In panel A, the result of an alternative model is shown as a dark blue solid-line, which is obtained for a reticulocyte/RBC preference ratio of 1:1 and fits the data better than the 477:1 ratio. Panel B: Time courses for the reticulocyte maturation time and erythropoietic output. Panel D: Time courses of the numbers of cells being lost due to the bystander effect and to parasitization. Panel E: Time courses of the numbers of cells being lost by senescent removal and random loss. Panel F: Experimental data for infected RBCs (green dots), model fit to the infected RBCs (green line), time course of immune response(purple line), and sub-curative and curative treatments windows (pink boxes, where the left side of each box depicts the experimental point-of-treatment, the window width depicts the modelled time course of progressive cell removal, until the right side of the box is reached, where the model assumes that all cells are killed by the treatment). Gray regions in all panels depict the period of time after which this macaque received a blood transfusion, which prevents its profile from being modelled.

From Day 10 on, the infection in RMe14 grew at an exponential rate equivalent to 11.4 merozoites released per infected RBC. Interestingly, RMe14 was the only macaque of his cohort with a parasite growth rate smaller than 20, the maximal biologically expected value. As the infection progressed, so did the number of RBCs consumed by the parasites (Fig. S2.1D), and this increase occurred concomitantly with loss of RBCs due to the bystander effect (Fig. S2.1D). Between Days 15-20, the infection growth slowed down, which the model suggested to be due to an increase in the strength of the immune response (Fig. S2.1F).

In stark contrast to RFa14, RMe14 developed severe anaemia, which mandated sub-curative treatment with artemether (1 mg/kg) on Day 20 and a blood transfusion on Day 23 [1]. Due to the blood transfusion, the inferred reticulocyte profile obviously does not match the experimental profile (Fig. S2.1A) from Day 22 onward. The model expects all RBCs produced to come from that same animal’s bone marrow, which means that every RBC had to have been a reticulocyte before maturing into a RBC. However, since this macaque received a blood transfusion, the RBC numbers went up without ever being reticulocytes in this same macaque, which explains the lack of fit in the reticulocyte profile (Fig. S2.1A).

Ultimately, this macaque recovered from its anaemia and, as in RFa14, the production of RBCs slowed down below its own baseline at Day 45, due, likely, to a decrease in senescent death caused by a shift in the age distribution of RBCs. Interestingly, the infection in this macaque also did not show evidence of a high reticulocyte preference over mature RBC, and the reticulocyte profile between Days 16 and 21 fitted better with an equal invasion probability of 1:1 for reticulocytes and RBCs.

**Modelling RSb14**

RSb14, like the other macaques, did not show any deviation from its initially healthy phenotype until the 7^th^ day post infection. These data points were used to establish its baseline of 24 hours of reticulocyte maturation time in circulation and of 2,900 RBCs/h/µL of erythropoietic output (Fig. S2.2B).

At Day 9, two days before the infection became patent, reticulocytes started to accumulate, which resulted in an elevated number of reticulocytes up to Day 14 (Fig. S2.2A). Given that this increase occurred without an increase in RBC numbers (Fig. S2.2C), it was modelled as an increase in reticulocyte maturation time, which was predicted to increase to 52 hours between Days 7 and 13 (Fig. S2.2B).

Between Days 15 and 21, the reticulocyte numbers exhibited oscillatory behavior, which was likely due to parasite invasion of reticulocytes. The reticulocyte preference that best fitted the reticulocyte profile in this macaque was 100:1 (shown in dark blue in Fig. S2.2A), whereas the reported preference of 477:1 [2] predicted a much lower level of reticulocytes (Fig. S2.2A).

Starting on Day 11, the parasitaemia grew at a rate equivalent to 27.3 merozoites released per infected RBC within a 48-hour life cycle. Unlike RFa14 and RMe14, this macaque was able to control the infection on its own, starting at Day 16, and did not require sub-curative treatment (Fig. S2.2F). The increase in parasitaemia was accompanied by RBC losses, both due to parasite invasion and to the bystander effect (Fig. S2.2D).


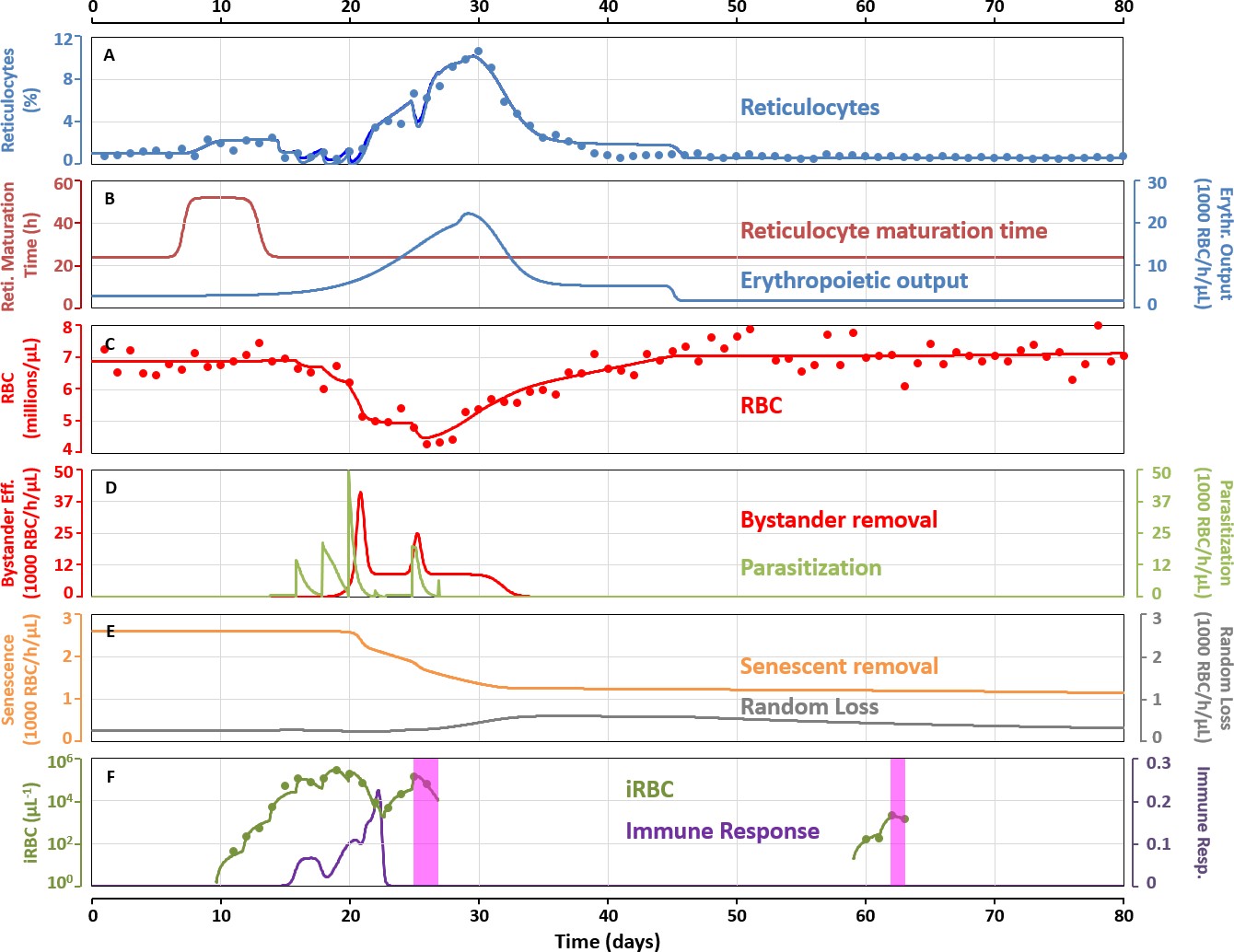


**Figure S2.2 Characterization of RSb14 infection.** Panels A, C and F show the experimentally obtained data for reticulocytes, RBCs, and infected RBCs, with the corresponding model fits. In panel A, the result of an alternative model is shown as a dark blue solid line, which is obtained for a reticulocyte/RBC preference ratio of 100:1 and fits the data better than the 477:1 ratio. Panel B: Time courses for the reticulocyte maturation time and erythropoietic output. Panel D: Time courses of the numbers of cells being lost by the bystander effect and by parasitization. Panel E: Time courses of the numbers of cells being lost by senescent removal and random loss. Panel F: Experimental data for infected RBCs (green dots), model fit to the infected RBCs (green line), time course of the immune response (purple line), and sub-curative and curative treatment windows (pink boxes, where the left side of each box depicts the experimental point-of-treatment, the window width depicts the modelled time-course of progressive cell removal, until the right side of the box is reached, where the model assumes that all cells are killed by the treatment).

Unlike any of the other macaques, up-regulation of the erythropoietic output occurred gradually while parasitaemia was high (Fig. S2.2B & F). By contrast, the peaks of erythropoietic output in other macaques tended to alternate between peaks of RBC loss, due to invasion and the bystander effect.

The reticulocyte numbers (Fig. S2.2A) between Days 39 and 48 seem to be over-estimated, but this level is predicted for a production of 5,290 RBCs/h/µL (Fig. S2.2B) that is required to sustain the increase in RBCs seen during this period (Fig. S2.2C).

After Day 45, production of RBCs dropped to 1,740 RBCs/h/µL (Fig. S2.2B), below this macaque’s baseline value, which was likely due to the lower senescent cell removal rate after the infection.

This macaque experienced a small relapse between Days 60 and 63. This relapse, however, cured itself by Day 62 leaving no evidence of an increased immune response. Moreover, this relapse did not have any haematological consequences, as the numbers of consumed RBCs and reticulocytes were so small that they did not affect the experimental or modelled data. Unlike the relapse in RIc14, this relapse was not preceded by an increase in reticulocytes.


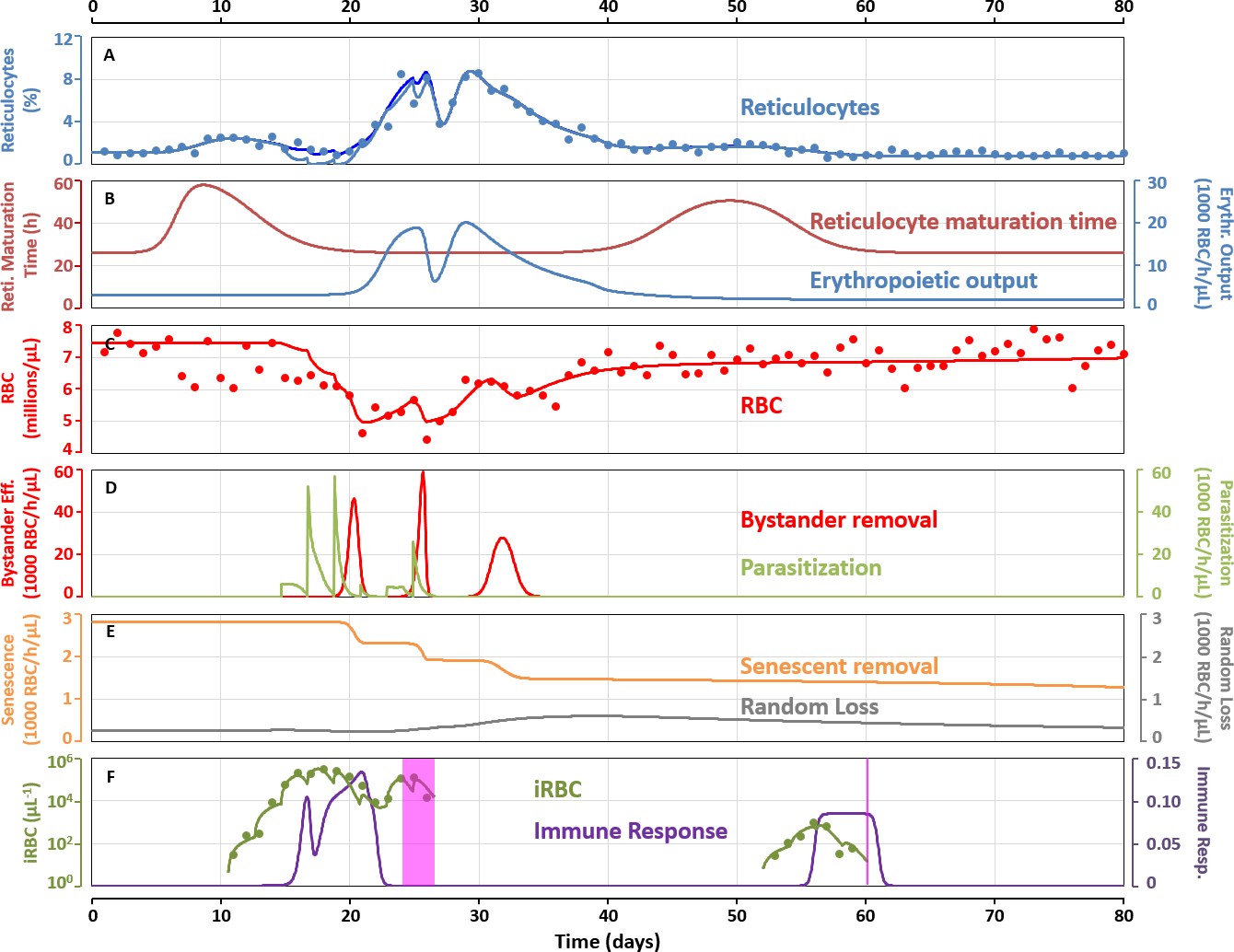


**Figure S2.3 Characterization of RIc14 infection.** Panels A, C and F show the experimental data for reticulocytes, RBCs, infected RBCs, along with the corresponding model fits. Panel A also shows the results of an alternative model as a dark blue solid line, which was obtained for a reticulocyte/RBC preference ratio of 10:1 and fits the data better than the 477:1 ratio. Panel B: Time-courses for the reticulocyte maturation time and erythropoietic output. Panel D: Time courses of the numbers of cells being lost by the bystander effect and by parasitization. Panel E: Time courses of the numbers of cells being lost by senescent removal and random loss. Panel F: Experimental data for infected RBCs (green dots), model fit to the infected RBCs (green line), time course of the immune response (purple line), and sub-curative and curative treatments windows (pink boxes, where the left side of the boxes depicts the experimental point-of-treatment, the window width depicts the modelled time-course of progressive cell removal, until the right side of the box is reached, where the model assumes that all cells are killed by the treatment).

**Modelling RIc14**

As with the other macaques, the infection profile for RIc14 remained at baseline for about four days. This time span allowed the calculation of the baseline values of 26 hours for the reticulocyte maturation time in circulation and an erythropoietic output of 3,140 RBCs/h/µL (Fig. S2.3B).

Reticulocyte numbers peaked between Days 6 and 15 (Fig. S2.3A), before parasitaemia became patent in the blood, which occurred at Day 11 (Fig. S2.3F). This transient accumulation of reticulocytes was modelled as a shift toward the release of younger reticulocytes, which increased their maturation time in circulation from 26 to 58 hours (Fig. S2.3B). Concomitant with the peaking of reticulocytes at Day 11 (Fig. S2.3A), the infection became patent and grew at a rate equivalent to 39.5 merozoites released per infected RBC. Between Days 15 and 20, parasitaemia levels became high and anaemia started to set in (Fig. S2.3C). Model prediction of reticulocyte destruction by parasites, based on the reticulocyte preference of 477:1 [2], was clearly overestimated (Fig. S2.3A), and the more conservative preference of 10:1, inferred from the profile, resulted in a much better fit.

After Day 20, the immune response peaked, and parasitaemia started to wane (Fig. S2.3F). This decrease may have been the cause for the up-regulation of erythropoietic output (Fig. S2.3B) which led to the improvement of the anaemic state (Fig. S2.3C). On Day 24, the parasitaemia regained strength, and two days later the RBC production rate fell. At this point the macaque received curative treatment [1] (Fig. S2.3F), which may have allowed the erythropoietic output to recover (Fig. S2.3B) and in turn resulted in recovery from the anaemic state.

Interestingly, the macaque still suffered some loss of RBCs at around Day 32 (Fig. S2.3C) which, given the absence of parasites, seemed to be attributable to the bystander effect (Fig. S2.3D). Moreover, this macaque experienced a relapse between Days 53 and 59, which was preceded by a peak of reticulocytes and modelled as a shift towards the release of younger reticulocytes. This shift was not as large as the first, peaking with a reticulocyte maturation time of 50 hours. The macaque was able to control the relapse on its own, and the infection cleared at Day 60 (Fig. S2.3F). As with other macaques, the erythropoietic output settled at a value below its baseline towards the end of the infection (after Day 50), due to the drop in the senescent loss of RBCs.

**References**

1. Joyner, C., Moreno, A., Meyer, E.V.S., Cabrera-Mora, M., Kissinger, J.C., Barnwell, J.W., Galinski, M.R.: *Plasmodium cynomolgi* infections in rhesus macaques display clinical and parasitological features pertinent to modelling vivax malaria pathology and relapse infections. Malaria Journal 15, 451 (2016). doi:10.1186/s12936-016-1480-6
2. Warren, M., Skinner, J.C., Guinn, E.: Biology of the simian malarias of southeast Asia. i. Host cell preferences of young trophozoites of four species of *Plasmodium*. The Journal of Parasitology 52, 14–6 (1966)
